# Liquid-liquid phase separation drives immune signaling transduction in cancer: a bibliometric and visualized study from 1992 to 2024

**DOI:** 10.3389/fonc.2025.1509457

**Published:** 2025-03-04

**Authors:** Yanhong Pei, Haijie Liang, Yu Guo, Boyang Wang, Han Wu, Zhijian Jin, Shanyi Lin, Fanwei Zeng, Yifan Wu, Qianyu Shi, Jiuhui Xu, Yi Huang, Tingting Ren, Jiarui Liu, Wei Guo

**Affiliations:** ^1^ Department of Bone Tumor, Peking University People’s Hospital, Beijing, China; ^2^ Department of Anatomy, Histology and Embryology, School of Basic Medical Sciences, Health Science Center, Peking University, Beijing, China; ^3^ Neuroscience Research Institute, Peking University, Beijing, China

**Keywords:** liquid liquid phase separation (LLPS), immune transduction, bibliometric, CiteSpace, cancer

## Abstract

**Background:**

Liquid–liquid phase separation (LLPS) is a novel concept that could explain how living cells precisely modulate internal spatial and temporal functions. However, a comprehensive bibliometric analysis on LLPS and immune signaling processes in cancer is still scarce. This study aims to perform a bibliometric assessment of research to explore the landscape of LLPS research in immune signaling pathways for cancer.

**Methods:**

Utilizing the Web of Science Core Collection database and multiple analysis software, we performed quantitative and qualitative analyses of the study situation between LLPS and immune signaling in cancer from 1992 to 2024.

**Results:**

The corresponding authors were primarily from China and the USA. The most relevant references were the “International Journal of Molecular Sciences”, “Proteomics”. The annual number of publications exhibited a fast upward tendency from 2020 to 2024. The most frequent key terms included expression, separation, activation, immunotherapy, and mechanisms. Qualitative evaluation emphasized the TCR, BCR, cGAS-STING, RIG-1, NF-κB signaling pathways associated with LLPS processes.

**Conclusion:**

This research is the first to integratively map out the knowledge structure and forward direction in the area of immune transduction linked with LLPS over the past 30 years. In summary, although this research area is still in its infancy, illustrating the coordinated structures and communications between cancer and immune signaling with LLPS within a spatial framework will offer deeper insights into the molecular mechanisms of cancer development and further enhance the effectiveness of existing immunotherapies.

## 1 Introduction

Cancer is distinguished by genomic instability resulting in the accumulation of gene mutations and structural alterations throughout tumor progression (1, 2). These genomic changes may generate tumor-derived antigens, which can be identified by the immune system as foreign antigens and then trigger the cellular immune response (3, 4). The immune system exhibits a crucial role in immune surveillance (5, 6), as immune cells from both the innate and adaptive immune system infiltrate into the tumor derived microenvironment and further modulate tumor growth and progression (7, 8). In particular, innate immune cells are involved in suppressing tumors by either directly eliminating cancer cells or initiating adaptive immune responses (8–10). As for the adaptive immune system, it is functionalized with T cells and B cells (5, 11, 12). The aforementioned immune systems have developed complex signaling networks to protect against pathogens or sterile threats. Nevertheless, cancer cells have developed multiple mechanisms, including deficiencies in antigen presentation machinery, the enrollment of immunosuppressive cell populations, and the upregulation of the negative signaling pathways (13–17).

Immunotherapy, which aims to bolster the body’s natural defenses to eradicate cancerous cells, stands as a significant advancement in cancer treatment, reshaping the landscape of oncology. While, various types of cancer have shown positive responses to immunotherapy (18–23),, the rates of response remain limited, and the underlying mechanisms are still elusive (24).Therefore, it is particularly important to explore the immune mechanism of cancer progression for cancer treatments.

Liquid-liquid phase separation (LLPS) is a cellular biological process wherein macromolecules spontaneously segregate into dilute and dense phases, forming bio-molecular condensates (25, 26). These compounds create a heterogeneous cellular microenvironment, specifically enhancing nucleic acids and proteins and exhibiting special features that promote biomolecule organization and concentration (27, 28). Anomalies in the separation of phases and transitions have been verified from liquid to solid in various neurodegenerative diseases (29). For instance, in a study conducted by Meng and colleagues, they discovered that Merlin (NF2) could induce the formation of phase-separated droplets when examining tissue samples extracted from individuals with vestibular schwannoma (30). However, growing research also suggests that altered LLPS plays a vital role in the phenotypes of cancer cells. It is proposed that cancer mutations could influence the ability of macromolecules to generate bio-molecular complexes, consequently impacting functionality indirectly. Additionally, bio-molecular condensates could serve as a formidable mechanism for spatial modulation in cancer cells (31), potentially explaining tumor heterogeneity and chemotherapy drug resistance by non-genetic theories. Despite the well-documented evidence of LLPS in fields such as transcriptional regulation and stress responses, there is still a lack of research on its relevance to immune signaling pathways.

The field of bibliometric utilizes both quantitative and qualitative analysis to study journals, publications, and their citation patterns, tracking changes over time and distribution trends within specific areas of interest, disciplines, institutions, and countries (32, 33). By employing bibliometric, researchers can pinpoint emerging research topics, plan out research directions, and forecast upcoming research trends (34). Co-citation and co-linearity methods are performed in bibliometric to identify the research foundation and highlight current research hotspots.

In this article, we use bibliometric analysis to detect the worldwide research trends between LLPS and immune signaling in cancer, and foresee potential future hotspots. Furthermore, we also provide an up-to-date insight of LLPS in driving the immune signaling pathway, including those triggered by TCR, BCR, cGAS-STING, and RIG-1 in cancer. As researchers focused on bone tumor disease, we have also reviewed the literature on LLPS and immune signaling related to bone tumors. However, there is no single literature that further elucidates the pathogenesis of bone tumor disease. Meanwhile, we discuss studies that have designed immunotherapy drugs involving LLPS process, and highlight some unresolved questions in the field of immunotherapy with LLPS. Hence, we conceive of the LLPS process as a promising strategy for cancer treatments. In general, understanding the role of LLPS in immune signaling transduction could reveal novel mechanisms of cancer progression and resistance, providing new targets for immunotherapy

## 2 Materials and methods

### 2.1 Search strategy

Recognized as a leading database platform, the Web of Science Core Collection (WoSCC) is known for its comprehensive coverage and authority. With a vast collection of over 12,000 international academic journals (35), it serves as a valuable source of global academic structure for R package “bibliometrix” software analysis, following the approach of previous studies (36–38).

**Figure 1:**
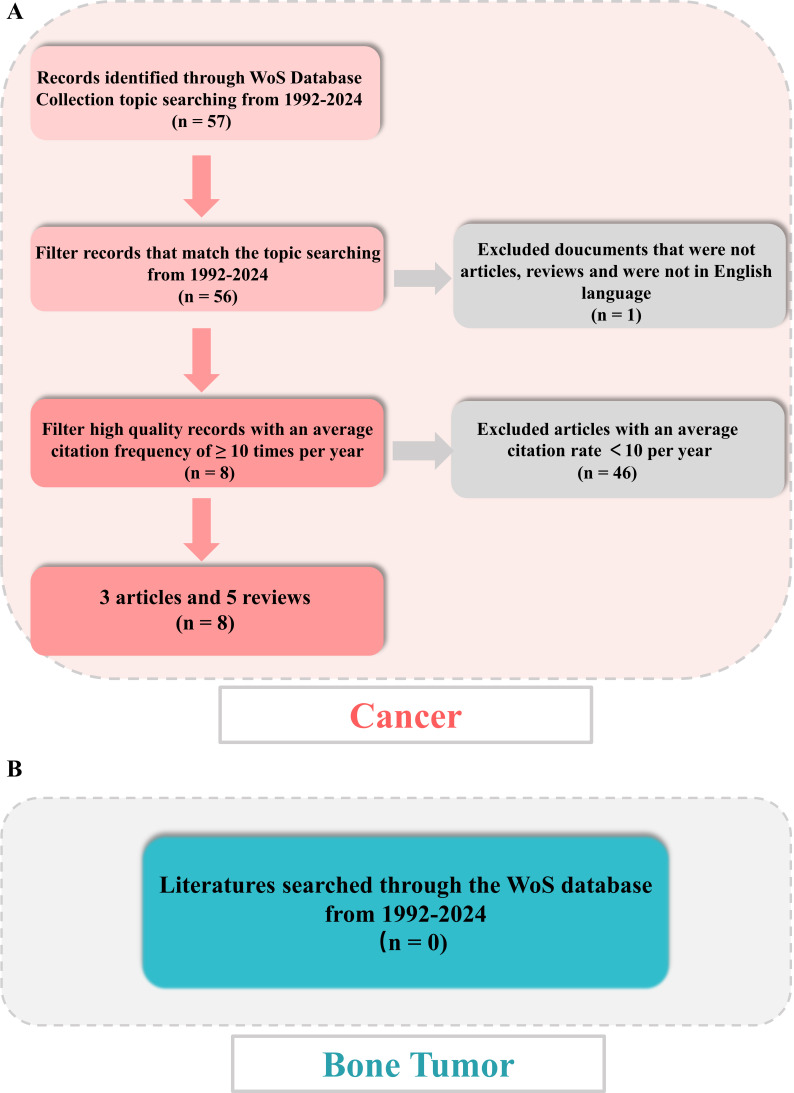
Flowchart of literature screening under bibliometric analysis. **(A)** Flow diagram in cancer. **(B)** Flow diagram in bone tumor.

Following the outlined procedure, all of the online literatures were extracted originated from the WoS database, covering period from January 1, 1992, to January 1, 2024. The search algorithm was as follows: TS = (tumor OR tumor OR cancer OR oncology) AND TS = (immune) AND TS = (liquid-liquid phase separation OR LLPS) AND publishing year = (1992-2024). The inclusion criteria for this study are as follows: (1) Peer-reviewed publications that primarily focus on the research field of LLPS and immune responses in tumor disease; (2) The document types must be either Article or Review; (3) The publications must be written in English; (4) The publication date must fall between 1992 and 2024. The exclusion criteria are as follows: (1) Publications that do not pertain to the themes of LLPS and immune responses in tumor disease; (2) literatures that are categorized as news, meetings, abstracts, briefings, etc. Then, obtained all valid data of literatures, including literature titles, authors, countries, institutions, abstracts, keywords, journals, and publishing years were stored in download_.txt files. The titles and abstracts of retrieved publications were independently screened by two reviewers, with any discrepancies being resolved through discussion with a third reviewer. Furthermore, both reviewers conducted a full-text review of all included references independently. A second full-text review was then performed on the discrepant articles to make the final decision for inclusion/exclusion. All disagreements were addressed by consulting with experts to reach a final consensus. Finally, all data were cleaned and analyzed individually by the co-authors and then cleaned separately using an R package. In addition, as part of the qualitative assessment, we further conducted a bibliometric screening of the literatures with an average annual citation ≥ 10. **Figure 1A** was the scheme of this study for cancer.

Meanwhile, for a deeper exploration of immune and LLPS, we further searched for literatures in bone tumor disease from 1992-2024, using the TS = (bone tumor OR bone cancer OR bone tumour) AND TS = (immune) AND TS = (liquid liquid phase separation OR LLPS) algorithm. The documents were presented in plain-text form and contained comprehensive citations for references to enhance the analysis and visualization by the R package “bibliometrix” software and CiteSpace software. **Figures 1B** (bone tumor) provided an in-depth summary of the data selected.

This study is a bibliometric article. Bibliometric articles typically do not require ethical approval because such studies do not involve direct experiments on humans or animals, but rather analyze and quantify research that has already been published.

### 2.2 Bibliometric analysis and visualization

The WoSCC database was utilized to analyze the fundamental characteristics of qualified literature, focusing on the number of publications and citations. The Relative Research Interest (RRI) was calculated as the ratio of publications in a specific field per year to the total literature across all fields. The world map was generated using the R software, which integrates numpy, python, matplotlib, and scipy. The publication timeline was created based on the method described in a previous article (39).

The H-index is an indicator of the impact of scientific research, reflecting a scholar’s publication of literatures that have been cited at least H times (40).

We utilized the VOSviewer (version: 1.6.20) to construct and visualize bibliometric networks. VOSviewer was used to analyze bibliometric coupling, co-occurrence, and co-citation in detail. Additionally, R package (version: 4.4.1) “bibliometrix” software was utilized to visualize publications among states, map international collaboration, and create a three-field plot analysis.

Furthermore, CiteSpace (version: 6.3.R1), developed by Professor Chen C, was utilized to construct a dual-map overlay for journals, to perform cluster-analysis of authors, institutions, nations and co-cited keywords, and to detect keywords and references with demonstrate citation bursts.

At last, we used the online website: http://www.bibliometric.com to further analysis the landscape of nations, affiliations, and authors according to the enrolled literatures.

## 3 Results

### 3.1 Main information of the published literatures

**Figure 2:**
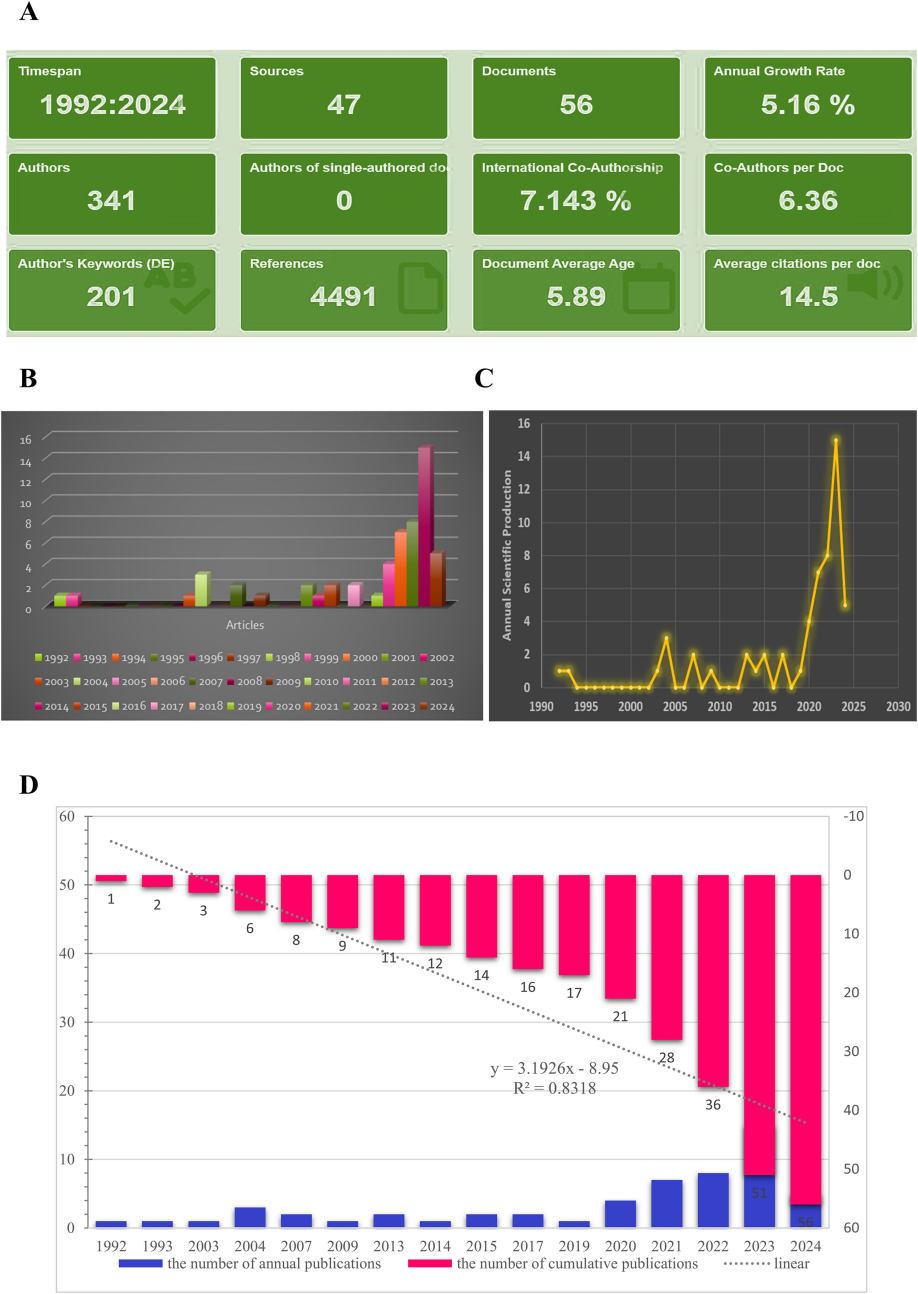
Main information. **(A)** Relevant literature from 1992-2024 under bibliometric analysis. **(B, C)** Graphs about the number of annual scientific production. **(D)** The number of cumulative publications from 1992-2024.

On January 1, 2024, a scientific literature search of the WoS was done to gain all online documents related to tumor, immune and LLPS. Based on the search criteria, a total of 57 literatures were gathered from 1992 to 2024, of which 43 articles (76.79%) and 13 reviews (23.21%) (**Figure 2A**).

As shown in **Figure 2A**, the annual growth rate was 5.16%, and the rate of international co-authorship was 7.143%. In addition, from 1992 to 2024, although only 1-2 literatures were published in the past nearly 30 years, the annual number of publications exhibited a fast upward tendency from 2020 to 2024 (**Figure 2B**, and 2C). The evolution trend of the cumulative number of productions followed the fitting curve *y* = 3.1926*x* – 8.95 (*R²* = 0.8318) (**Figure 2D**), indicating that LLPS has become a progressively prominent research area for scientists and may represent an enduring and promising field of study.

### 3.2 Analysis of countries and institution

**Figure 3:**
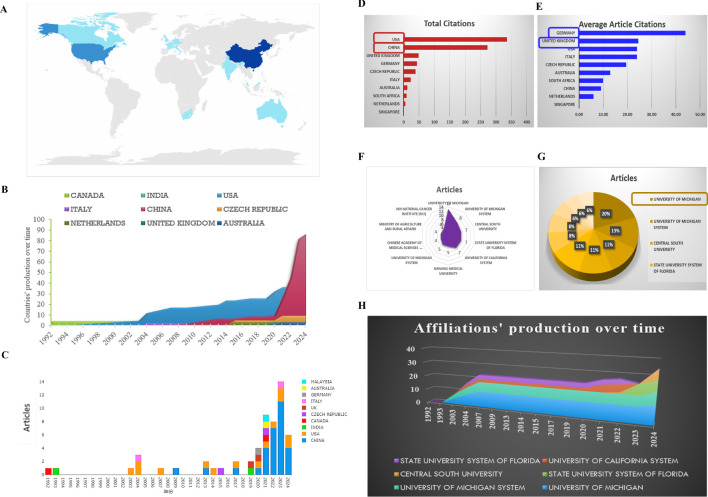
Analysis of the distribution over the world. **(A)** Country’s scientific production. **(B)** Country production over time. **(C)** Column chart exhibiting the countries production over time. **(D)** Most cited countries. **(E)** Average cited countries. **(F, G)** Most relevant affiliations exhibited by radar map and pie chart. **(H)** Affiliations’ production over time.

The corresponding authors were primarily from China, the USA and the Czech Republic (**Figure 3A**). As depicted in **Figure 3B**, China is the nation that has published the largest number of literatures, compared to other states. The number of literatures linked to China presented a rapid and consistent increase over time, contrasting with the USA, where the increase was more moderate (**Figures 3B, C**).

The USA (total citations: 336) and China (total citations:273) are the main nations with the most total citations, demonstrating the superior quality of their articles in this research field (**Figure 3D**). While, for the average article citations, Germany reaches the highest rank (44.00%, ranks 1), followed by the United Kingdom (24.50%, ranks 2), the USA (24.00%, ranks 3), ITALY (24.00%, ranks 3) (**Figure 3E**). According to the total citations and the average article citations, a deep reflection should be needed as a member of the Chinese scientific researcher.

A filled radar chart, also known as a solid radar chart, is a visual representation that uses filled areas to show data points in a multi-dimensional space. This chart includes multiple axes, each representing a different variable or category, radiating from a central point like the spokes of a wheel. Each data point is plotted on the chart using coordinates on the axes, with the distance from the center indicating the value for that category. The data points are connected to create a closed shape, which can be filled with color or shading to show the overall performance or value across all categories. Points that are further from the center represent higher values, while points closer to the center indicate lower values. As shown in **Figures 3F**, 3G and 3H, the University of Michigan (14, 20%), the University of Michigan system are the primary affiliation of these studies, followed by the Central South University, the state university system of Florida and University of California system. Regrettably, the Chinese Academy of Medical Sciences - Peking Union Medical College ranks last, not containing Peking University.

At last, by the online website (http://www.bibliometric.com), top 10 for author’s impact was exhibited in **Table 1**.

**Table 1:** Top 10 for institution’s impact.

Affiliation	Articles	Total Citation	Average Citation	Number of the first author	Total citation of the first author	Average citation of the first author
Zhejiang Univ	3	12	4.00	2	6	3.00
Chinese Acad Med Sci	4	7	1.75	1	0	0.00
Cent South Univ	10	6	0.60	3	3	1.00
Nanjing Med Univ	8	6	0.75	3	2	0.67
NHC Key Lab Combined Multi Organ Transplantat	1	6	6.00	0	0	0.00
Res Ctr Diag & Treatment Hepatobiliary Dis	1	6	0.75	0	0	0.00
Zhejiang Shuren Univ	1	6	6.00	0	0	0.00
Huazhon Univ & Technol	5	4	6.00	3	2	0.67
Hunan Key Lab Translat Radiat Oncol	1	3	6.00	0	0	0.00
Sun Yat Sen Univ	1	3	3.00	1	1	1.00

### 3.3 Author analysis

**Figure 4:**
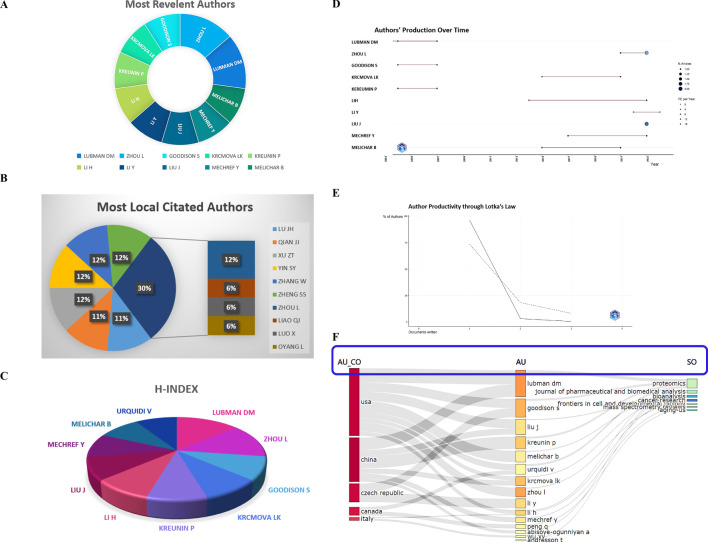
Authors analysis. **(A)** Most relevant authors. **(B)** Most local cited authors. **(C)** The H-index with authors. **(D)** Authors’ production over time. **(E)** Authors’ productivity through Lotka’s law. **(F)** The correlation of the states (left), authors (middle), and journals (right) based on the alluvial flow map under R package in cancer.

Lubman DM (articles: 3, articles fractionalized: 0.62), is closely followed by Zhou L (3, 0.37), Goodison S (2, 0.45), Krcmova LK (2, 0.27), Kreumin P (2, 0.45), Li H (2, 0.15), Li Y (2, 0.27), Liu J (2, 0.17), Mechref Y (2, 0.30), and Melichar B (2, 0.27) as the most relevant authors with fractionalized articles, as shown in **Figure 4A**. Furthermore, all of these researchers have consistently authored highly cited studies every year

The local citations of the researchers were measured, with Lu JH, Qian JJ, Xu ZT, Yin SY, Zhang W, Zheng SS, and Zhou L each having 6 local citations, as exhibited in **Figure 4B**. Other authors had an average of 3 local citations.

The H-index and the author’s production over time were shown in **Figure 4C, D**, and the authors’ productivity through Lotka’s law was presented in **Figure 4E**. Additionally, a cluster analysis of cooperative institutes was performed, revealing that the USA researchers predominantly published in the “Proteomics”, “Journal of Pharmaceutical and Biomedical Analysis”, “Bioanalysis”, and “Cancer Research” journals, while authors originated from China tended to publish in the “Frontiers in Cell and Developmental Biology” and “Cancer Research” journals (**Figure 4F**). At last, by the online website (http://www.bibliometric.com), the top 10 authors’ impact was exhibited in **Table 2**.

**Table 2:** Top 10 for author’s impact.

Authors	Articles	Total Citation	Average Citation	Number of the first author	Total citation of the first author	Average citation of the first author	Number of the corresponding author
LI, YL	1	0	0.00	1	0	0.00	0
PENG, Q	2	3	1.50	1	3	3.00	1
WANG, LJ	1	0	0.00	0	0	0.00	0
TAN, SM	1	3	3.00	0	0	0.00	0
XIA, LZ	1	3	3.00	0	0	0.00	0
WU, NY	1	3	3.00	0	0	0.00	0
QYANG, L	1	3	3.00	0	0	0.00	0
TANG, YY	1	3	3.00	0	0	0.00	0
SU, M	1	3	3.00	0	0	0.00	0
LUO, X	1	3	3.00	0	0	0.00	0

### 3.4 Source analysis

**Figure 5:**
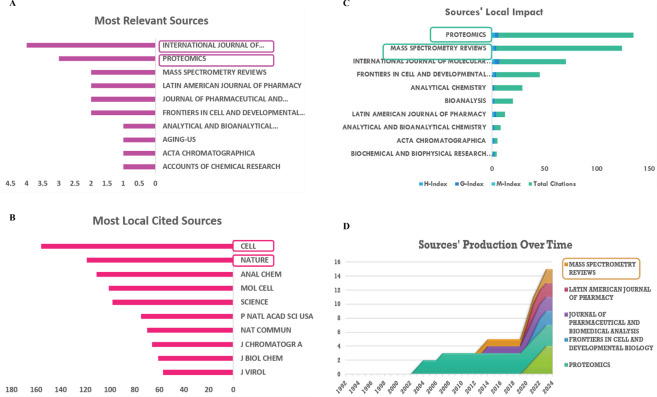
Analysis of sources. **(A)** Most relevant sources. **(B)** Most local cited journals. **(C)** Sources’ local impact. **(D)** Sources’ production over time.

The most relevant references were obtained in the journals “International Journal of Molecular Sciences” (articles: 4), “Proteomics” (articles: 3), and “Frontiers in Cell and Developmental Biology” (articles: 2), which are leading publications in the field of immune and LLPS in cancer (**Figure 5A**). The most locally cited sources are mainly in the journals “Cell”, “Nature”, “Analytical Chemistry”, “Molecular Cell”, “Science” and “Nature Communications” (**Figure 5B**). Moreover, according to **Figure 5C**, the primary journals where the key sources contributing to the local impact were published include “International Journal of Molecular Sciences (H-index: 3)”, “Proteomics (H-index: 3)”, and “Frontiers in Cell and Developmental Biology (H-index: 2)”. Furthermore, it is worth noting that, in the field of cancer, the relevant literature between immune and LLPS started to emerge in 2002, as indicated in **Figure 5D**. Lastly by the online website (http://www.bibliometric.com), the top 10 authors’ impact was exhibited in **Table 3**.

**Table 3:** Top 10 for journal’s impact.

Journal	Articles	Total Citation	Average Citation
FRONTIERS IN CELL AND DEVELOPMENTAL BIOLOGY	2	7	3.50
INTERNATIONAL JOURNAL OF BIOLOGICAL SCIENCES	1	3	3.00
JOURNAL OF TRANSLATIONAL MEDICINE	1	2	2.00
FRONTIERS IN ONCOLOGY	1	1	1.00
BMC CANCER	1	1	1.00
CANCER RESEARCH	1	1	1.00
JOURNAL OF CLINICAL LABORATORY ANALYSIS	1	1	1.00
ELECTROPHORSISI	1	1	1.00
BIOANALYSIS	1	1	1.00
AGING-US	1	0	0.00

Thus, selecting an appropriate journal is essential for scientists, as it enables them to align their research objectives with the specific aims and scope of the journal. This alignment is crucial to establish a solid and strong theoretical basis for the study of immune and LLPS, especially in the field of cancer.

### 3.5 Cited document analysis

**Figure 6:**
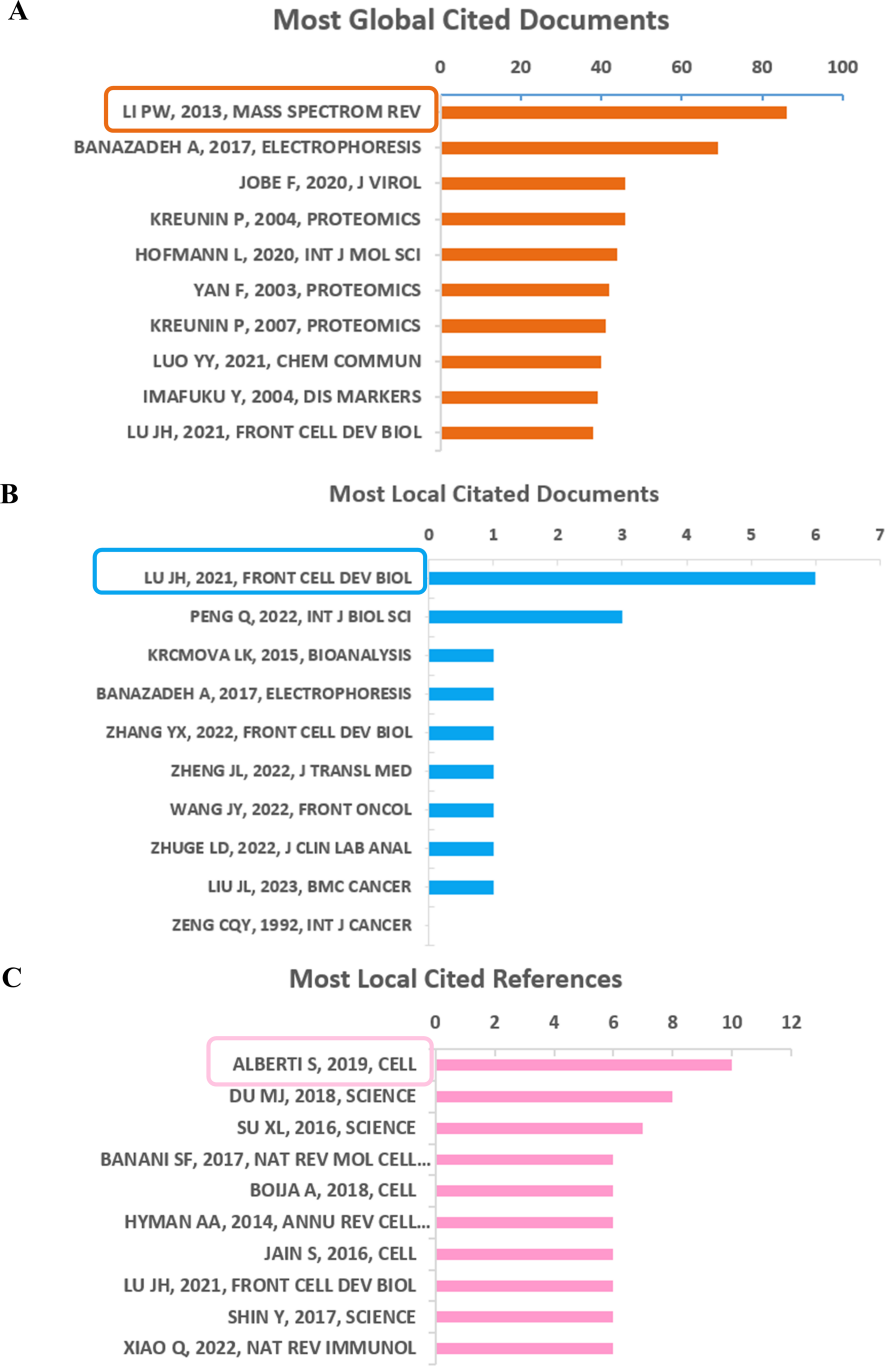
Analysis of documents based on R package. **(A)** Most global cited documents. **(B)** Most local cited documents. **(C)** Most local cited references.

The most globally cited document was “Advanced hyphenated chromatographic-mass spectrometry in mycotoxin determination: current status and prospects” (total citations: 86), which was published online in the journal “Mass Spectrom review” by Li PW, et al., in 2013. The ranked 2 (total citations: 69) was published in “Electrophoresis” with the title “Recent advances in mass spectrometric analysis of glycoproteins” by Banazadeh A., et al., in 2017. Tied for the third most globally cited were, “Respiratory Syncytial Virus Sequesters NF-κB Subunit p65 to Cytoplasmic Inclusion Bodies To Inhibit Innate Immune Signaling” (total citations: 46), published in “Journal of Virology” by Jobe F, et al., in 2020 and “Identification of metastasis-associated proteins in a human tumor metastasis model using the mass-mapping technique” (total citations: 46), published in the journal “Proteomics” by Kreunin P, et al., in 2004 (**Figure 6A**).

Furthermore, the most frequently referenced documents within the local community were “Emerging Roles of Liquid-Liquid Phase Separation in Cancer: From Protein Aggregation to Immune-Associated Signaling” (local citation = 6), which was authored by Lu JH, et al., and published in “Frontier in cell and developmental biology” in 2021. Following closely behind (local citation = 3) was “Phase Separation in Cancer: From the Impacts and Mechanisms to Treatment Potentials” in the journal “International Journal of Biological Sciences” by Peng Q, et al., in 2022 (**Figure 6B**).

However, the most locally cited references were Alberti S, 2019, Cell; Du MJ, 2018, Science; Su XL, 2016, Science; Banani SF, 2017, Nat Rev Mol Cell, as shown in **Figure 6C**.

### 3.6 Analysis of the key words and trend topics

**Figure 7:**
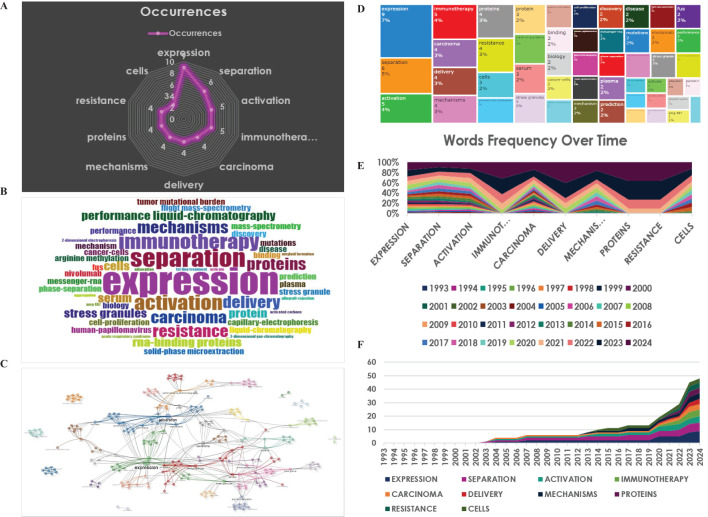
Analysis of key words. **(A)** Radar map for the most frequent words. **(B)** World-Cloud for the relevant words **(C)** Thematic map under the clustered analysis. **(D)** Tree-Map for the relevant words. **(E, F)** Words frequency over time.

The most frequent key terms included expression, separation, activation, immunotherapy, carcinoma, delivery, mechanisms, proteins, resistance, and cells. These mentioned key words are also displayed in a plot chart, a word cloud, and a tree map, with their size reflecting the significance and frequency (**Figure 7A–C**).

Moreover, trend topics were depicted in **Figure 7D, E**, exhibiting that separation was the most researched topic in the nearly past 10 years, while immunotherapy has been the hotspot and the primary area of study in the recent 10 years (**Figure 7D, E**). This subtle shift indicated a growing interest in exploring the potential links between immunity and LLPS in cancer therapy (**Figure 7F**).

### 3.7 Cluster analysis of the authors, affiliations, and states

**Figure 8:**
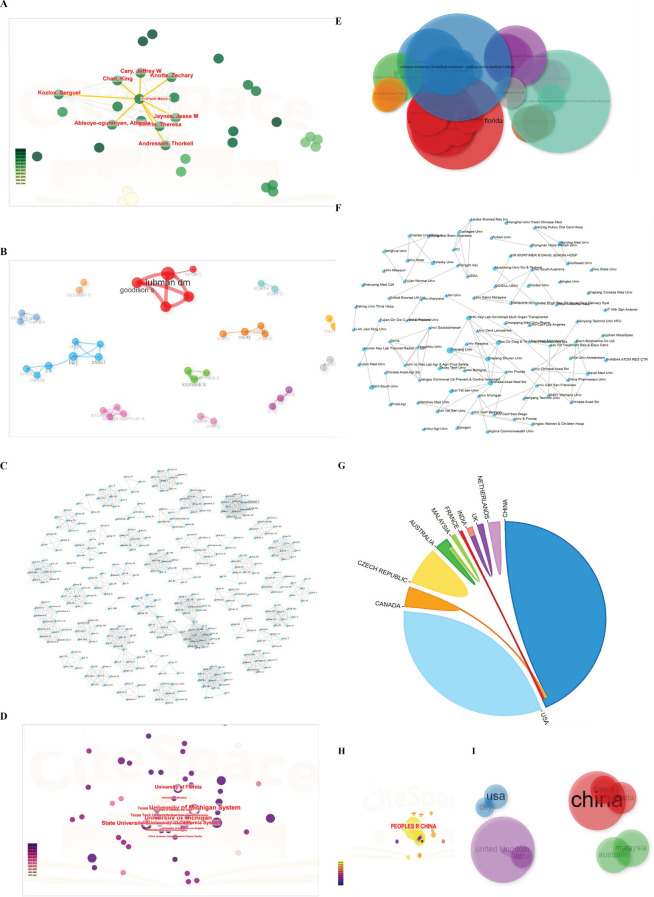
Cluster analysis of authors, affiliations, collaborated nations. **(A)** Cluster analysis of authors by CiteSpace software. **(B)** Cluster analysis of authors by Bibliometric software. **(C)** Cluster analysis of authors under http://www.bibliometric.com. **(D)** Cluster analysis of affiliations by CiteSpace software. **(E)** Cluster evaluation under bibliometric software based on R package. **(F)** Cluster analysis of collaborated institutions by http://www.bibliometric.com. **(G)** Cluster analysis of collaborated nations by http://www.bibliometric.com. **(H, I)** Cluster analysis of states under CiteSpace software and bibliometric software.

Then, a total of 57 documents were analyzed for collaborations between authors, institutions, and countries using the CiteSpace visualization. As shown in **Figure 8A**, Jaynes, Jesse M, Abisoye-ogunniyan, Abisola, Cray, Jeffrey W, Chan King, Knotts, Zachary, Kozlov, Serguel, O’neill Martinic, and Andresson, Thorkell worked closely with one another. Furthermore, by the bibliometric analysis, the collaboration network for the authors: Lubman DM, Goodison S, Barder TJ; Zhou I, Liu J, Cang S, Li H, Chen CS, Byrd JC, as exhibited by the **Figure 8B**. Additionally, we also conducted the cluster analysis of authors using the online website at http://www.bibliometric.com. As shown in **Figure 8C**, there were numerous co-authors, which indicated that the online analysis bibliometric website is greater than the other software.

Moreover, as depicted in **Figures 8D-F**, the University of Michigan, the State University System of Florida, the University of California System, the Texas Tech University System and the Texas Tech University had a strong collaboration with each other. However, interestingly, other institutions did not have collaborations on account of just a few researches about the immune and LLPS. The cooperation among nations predominantly occurred from China, Canada, and France, not containing the USA, demonstrating the necessity and the importance of enhancing global teamwork (**Figures 8G-I**).

The above analysis results suggested that potential collaborations or funding opportunities that could help bridge this gap.

### 3.8 Analysis of the co-appearance network of the key words

The quantity and frequency of keywords appearing during a specific timeframe are essential for evaluating the current and future advancements in a specific field of research. Following that, we utilized CiteSpace software and VOSviewer software to perform a co-occurrence network analysis of the key words, and the outcomes were depicted through a visualization graph, cluster photo, and timeline chart.

**Figure 9:**
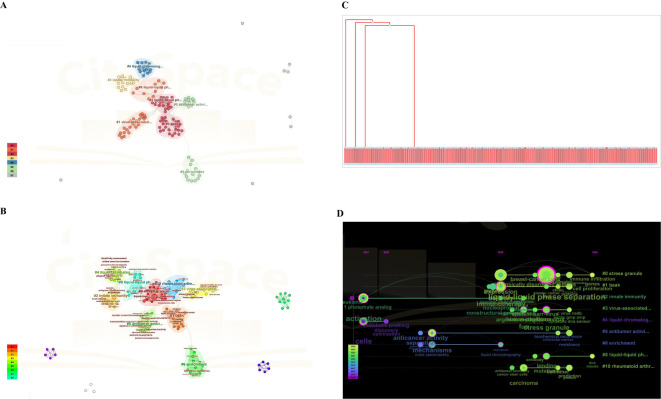
Cluster analysis of key words using CiteSpace software and VOS viewer. **(A)** Analysis of VOSviewer. **(B)** Co-occurrence network analysis of keywords based on VOSviewer and CiteSpace software. In the visualized graph, keywords are divided into 8 clusters with different colors. **(C)** Trend topics diagram under R package. **(D)** The timeline of keywords based on CiteSpace software and VOS viewer.

**Figure 9A** displays the most frequently appearing keywords, which align with the presented in **Figure7B**. **Figure 9B** is the cluster photo and timeline chart, respectively, indicating immunotherapy, solid phase microextraction, antitumor activity, protein, tumor-associated, liquid chromatography, disease, enrichment, autophagy, nuclear import, and stress gauge. The focus of research constantly altered towards immunotherapy from separation, as depicted in **Figure 9C**. As depicted in **Figure 9C**, the trend topics diagram under the R package was so little because of the searched literature was scarce. The timeline of keywords presented that the focus of research gradually shifted towards immunotherapy and LLPS based on CiteSpace software and VOSviewer (**Figure 9D**), which indicated that this shift may be related to the fact that the prospects for cancer treatment have turned towards tumor immunotherapy as a fourth-line treatment.

### 3.9 Quantitative analysis of the literature enrolled

After implementing the Bibliomtrix filter with an average citation of ≥ 10 per year, 8 documents were identified. Subsequently, a qualitative assessment was performed. This evaluation aimed to not only elucidate the current research progress but also to gain a deeper level of comprehension for the intricate relationship between immune and LLPS in cancer. Multiple receptors from the surface of immune cells, in conjunction with ligands or/and downstream binding partners, can cluster ranging from nanometers to micrometers on the plasma membrane (41–43).

How do LLPS condensates affect cell functions in the physiological and pathological environment? Speculatively speaking, as liquid-like condensates progress into gel-like structures, they may potentially transmit force much more effectively. Numerous immune receptors (just like BCR, low-affinity IgG receptor FcγRIIA, and TCR), are known to be sensitive to force (44–46). This demonstrates that the shift in the properties of condensates might modulate the activation of receptors.

Here, we would elaborate on how LLPS influences the immune signaling cascades by regulating these membrane clusters but many fields still require investigation.

#### 3.9.1 LLPS on the plasma membrane

##### 3.9.1.1 TCR (T cell receptor) signaling pathway

A predominant characteristic of the TCR signaling pathway is the formation of distinct clusters (nearly discontinuous micrometer- or submicrometer sized) on the plasma membrane. Since the 1990s, scientific researchers have constructed a variety of groups that form microclusters, including TCR, CD28, and PD1; kinases such as ZAP70 and LCK; the enzymes like PLCγ1, CBL, and SOS1; the adaptor proteins like SLP76, GADS, LAT, and GRB2 (47–49). Microcluster formation of T cell is highly dependent on the ligand binding and phosphorylation. Previous studies have indicated that SOS1, GRB2, and LAT are are pivotal oligomeric components in the generation of T cell microclusters (50).

LAT microclusters exhibit properties similar to liquids and are created through LLPS of LAT and its binding partners (51). Notably, SOS1 and PLCγ1,the two enzymes, also play a scaffolding role in enhancing LAT cluster formation in an enzyme-independent manner (52, 53). LAT condensates could facilitate tyrosine phosphorylation, a crucial marker for the activation of the TCR signaling pathway, by concentrating kinases, not excluding phosphatases in the clusters.

Despite the relatively well understood LAT microclusters, the potential mechanism of transmembrane receptor clusters (like TCR, CD28, and PD1) remains unclear. Additionally, the properties of these receptor clusters and the extent of LLPS in driving these TCR cluster formations is still uncertain and need to be further investigated. Besides, another intriguing physiological phenomenon also could be interpreted by the LLPS biophysical process in the TCR signaling pathway. As we know, a notable aspect of the TCR signaling pathway is its capability to distinguish between self and non-self antigens, although the mechanisms of which are not yet comprehended (54, 55). Despite only a slightly fold difference for antigens, the signaling response is binary. LLPS may offer a compelling rationale for this phenomenon as it is a well-coordinated and collective process that leads to a binary result. Even a minor alteration in input, like a slight enhancement in the antigen-TCR interaction affinity, could induce phase separation and initiate the subsequent signaling pathway (56). Longhui Zeng, et al. unveiled a crucial function of PLCγ1(phospholipase Cγ1, PLCγ1) in facilitating the LAT LLPS and the activation of TCR signaling transduction (53).

##### 3.9.1.2 BCR signaling pathway

Protein SLP5 (called BLNK) triggers LLPS in the BCR signaling pathway. Some researchers have suggested that SLP65 could form a liquid-like complex by interacting with CIN85 (called SH3KBP1) via a traditional multivalent interaction involving the proline-rich motifs of SLP65 and the SH3 domains of trimeric CIN85 (57, 58).

It has been reported that SLP65 compounds are pre-existing in the cytoplasm of resting B cells, which is different from the time that LAT condensates formed (59). Meanwhile, in the process of condensates formed, the liposomes also are essential in facilitating condensates at physiological cellular concentrations of CIN85 and SLP65. Barbara L. Kee revealed groundbreaking activity of EBF1 as a pioneering transcription factor in B lymphocyte specification by recruiting the nucleosome remodeler Brg1 and driving the occurrence of FUS LLPS (60).

#### 3.9.2 Innate immune receptors

After binging with ligand, a multitude of innate immune receptors would cluster at the cell surface such as the mast cell receptorFcϵRI80-82 and phagocytic receptors-Drosophila melanogaster Draper and dectin 1 (61, 62), which are similar to the above mentioned BCR and TCR signaling clusters on accounting of the existence of immune receptor tyrosine-derived activation motifs (ITAMs) or ITAM-related sequences, micrometer/nanometer in size, and multivalent proteins that amplify signaling pathway transduction.

#### 3.9.3 Liquid-like condensates in immune cells

LLPS not only associates with signaling transduction of the plasma membrane but is also involved in the regulation of intracellular immune signaling pathways. This includes the retinoic acid-inducible gene I (RIG-I) pathway, the cyclic GMP–AMP synthase (cGAS)–stimulator of interferon genes (STING) pathway, and the nuclear factor-κB (NF-κB) pathway (56).

##### 3.9.3.1 cGAS liquid-like condensates

cGAS could detect abnormal cytosolic dsDNA originating from nuclear or mitochondrial injuries or pathogens (63). Then, dsDNA binds cGAS and activates it, synthesizes the compound of 2′3′-cyclic GMP–AMP (cGAMP), and then triggers the STING signaling pathway, resulting in the expression of pro-inflammatory cytokines and type I interferons (63, 64). Recent research has revealed that dsDNA binding to the cGAS could induce the production of liquid-like condensates related to LLPS (65).

K432T and G303E, the two tumor-related mutations, located at DNA-binding sites of cGAS, lead to a diminished capability to form cGAS condensates and a decrease in cGAMP (30).

Furthermore, investigations are necessary to be acquired to comprehend how these cGAS mutants shape immune responses as tumor progression. Additionally, manipulating cGAS condensate could offer a novel insight into regulating immune response against tumor. Wuchang Zhang et al. reported that the inhibition of KDM4A mechanistically promoted the formation of liquid-like HP1γ puncta on heterochromatin, halted DNA replication, and subsequently triggered the activation of cancer cell-intrinsic cGAS-STING signaling transduction (66). Fansen Meng et al. demonstrated that inducing the LLPS of mutant NF2 (Neurofibromin 2, NF2) could result in quiescence of cGAS-STING signaling in antitumor immunity (30).

##### 3.9.3.2 RIG-I signaling pathway

A recent preprint demonstrated that RNA binding induces LLPS of TRIM25, recruits RIG-1 to condensates, boosts the ubiquitylation of TRIM25 (67). Conversely, RNF125 has been reported to inhibit the RIG-1 signaling pathway by enhancing the K48-linked ubiquitylation and degradation of RIG-1 (67–69).

##### 3.9.3.3 NF-κB signaling pathway

Fatoumatta Jobe has verified that viruses can prevent innate immune response by trapping the NF-κB subunit p65 in bio-molecular condensate (70). Ziran Qin has reported that in innate antiviral immunity, the LLPS of IRF3 and IRF7 were enabled by deacetylation with SIRT1 (71). In this section, our emphasis was on the activation of signaling cascades. However, there is also evidence demonstrating that PD1 could form microclusters upon engagement with PD-L1 (48). Nevertheless, it also remains unclear whether PD1 microclusters originated from LLPS. So, we would not discuss this situation here. Additionally, in the area of tumor immune infiltration, Yanling Li, et al. have found that EphA2 (Erythropoietin-producing hepatocellular A2, EphA2) could be involved in the advancement of colorectal cancer by forming LLPS condensates and further affecting the immune cell infiltration (72).

To summarize, the research on how LLPS relates to the immune signaling pathway remains still limited. While LLPS, as the biophysical process, has been verified to regulate the immune signaling pathways, just like TCR, BCR, cGAS, RIG-1, and NF-κB. Undeniably, this field is still emerging and needs to be further investigated for the functions of manipulating the immune system, including the aspects of detecting techniques and physiological studies.

#### 3.9.4 The regulated effect of LLPS-associated with immune responses in bone tumor disease

As researchers devoted to elucidating the mechanisms of bone tumor disease, we also searched relative literatures on the topics: LLPS, immune, and bone tumor. Surprisingly, there is no literatures, which indicated that research in the field of immunity related to LLPS is quite rare in orthopedic research.

We know that OS is an extremely rare malignant bone cancer, which commonly occurs in children and adolescents. The diagnosis and treatment of rare diseases is a long process, requiring a professional medical team and a lot of clinical experience. In addition, the treatment of children and adolescents is very different from that of adults, because children have a long way to go in the future, and doctors need to consider the long-term effects of medication, dosage, and treatment, including future rehabilitation planning, and even life planning.

In general, based on the above research background, we think that modulating the LLPS process could be a promising approach for OS disease, especially in the biological process of the immune system. One powerful strategy to develop chemotherapy drugs tailored towards LLPS condensates by targeting specific proteins has already been verified (73–75), which could allow for the selective targeting of abnormal LLPS without the harmful effects of broader chemotherapy treatments.

## 4 Discussion

### 4.1 Role of LLPS in cellular function

In the landscape of physical chemistry, phase separation is a familiar phenomenon. However, for many biological scientists and immunologists, this concept is not entirely understood yet. It refers that the biomolecules shift from a uniform microenvironment into two separate phases (the dilute phase and the condensed phase), where the movement and concentration of solutes vary dramatically (56). LLPS is commonly observed in cells due to the properties of the fluidic and aqueous environment of the intracellular space. The condensed phase often exchanges materials with the dilute phase while in a liquid-like state, and this characteristic is significant in shaping the composition and activity of molecules in the condensed phase (56). Due to the cellular condensates are often formed by various interactions, it is possible to observe a spectrum of intermediate states between liquid and solid forms. LLPS condensates are involved in various of biological functions, including organizing higher-order chromatin, sorting misfolded proteins, modulating gene expression, setting signaling clusters, and building cytoskeletal networks utilizing actin and microtubules (76). Of course, they also help in segregating cell fate determinants symmetrically and creating signaling assemblies in pre- and postsynaptic densities.

The condensates produce a heterogeneous cellular environment, which specifically enriches nucleic acids and proteins, and then further facilitate the concentration of biomolecules and organization (27, 28). The formation of interaction networks that involve multivalent proteins or nucleic acids plays a crucial role in LLPS and is primarily boosted by peptides with intrinsically disordered regions (IDRs), folded modular domains, or polymerizing domains (77).

### 4.2 LLPS in cancer progression

Moreover, it has been indicated that a majority of cell signaling proteins, as well as a significant number of cancer-derived proteins, contain extensive intrinsically disordered regions (IDRs), that are essential for driving LLPS process (78). Proteins associated with cancer and cancer-related mutations could regulate the quantity and assembly of condensates by affecting LLPS, which in turn ultimately drives the abnormal cellular activities and boosts the progression of tumorigenesis (**Table 4**) (79–82). Notably, irregular or abnormal transitions of condensates to a solid state are linked to some specific neurodegenerative diseases (including FUS (83, 84), TDP-43 (85, 86), HNRNPA1 (87), and DDX (88), as well as Tau (89, 90),) and cancers (such as transcriptional condensates, PRC1 condensates, super enhancers, DNA repair condensates, stress granules, Paraspeckles, SPOP/DAXX bodies and PML foci (91–96).

**Table 4:** Proteins condensates associated with LLPS progress in cancer.

Protein	LLPS condensates	Location	Role of LLPS in tumor disease
EphA2	EphA2 condensates	Cytomembrane	Associated with ferroptosis and immune cell infiltration in colorectal cancer{Liu, 2023 #215}
PLCγ1	LAT complex	Cytomembrane	PLCγ1 in promoting phase separation of the LAT complex and TCR signal transduction{Zeng, 2021 #216}
KDM4A	KDM4A complex	DNA replication stress	KDM4A activated tumor cell-intrinsic immunity by inducing heterochromatin compaction and replication stress{Zhang, 2021 #217}
53BP1	Nuclear complex	Nucleus	Hyper-assembly of 53BP1 on chromatin lead to LLPS impair cell survival in cancer{Ghodke, 2021 #219}
EBF1	Mediating recruitment of the nucleosome remodeler Brg1 and FUS-assisted liquid-liquid phase separation	Cytoplasm	Regulating the development and progress of cancer{Zolotarev, 2022 #218}
BRD4	Nuclear complex	Nucleus	BRD4's participation in super-enhancers is crucial for oncogene transcriptional dependency and the survival of cancer cells{Donati, 2018 #220}
YBX1	CircRNA-YBX1 complex	Cytoplasm	Cytoskeleton remodeling mediated by circRNA-YBX1 phase separation suppresses the metastasis of liver cancer{Liu, 2023 #221}
DAXX	Nuclear complex	Nucleus, Cytoplasm	SPOP/DAXX bodies formed via LLPS is important in inducing cancer cell apoptosis{Cai, 2021 #222;Mahmud, 2019 #223}
SPOP	Nuclear complex	Nucleus, Cytoplasm	SPOP/DAXX bodies formed via LLPS is important in inducing cancer cell apoptosis{Cai, 2021 #222;Mahmud, 2019 #223}
NONO	Paraspeckle	Nucleus	Impact on the tumor stability to develop drugs resistance{Pisani, 2020 #224}
SFPQ	Paraspeckle	Nucleus, Cytoplasm	Impact on the tumor stability to develop drugs resistance{Pisani, 2020 #224}
YTHDF1	P-body; cytoplasmic stress granule; neuronal ribonucleoprotein granule	Cytoplasm	1. P-bodies modulation of mRNA metabolism plays a critical factor in the development and progression of cancer{Nsengimana, 2022 #225} 2. Stress granules assembly is increased in tumor{Shi, 2019 #226}
YTHDF2	P-body; cytoplasmic stress granule; neuronal ribonucleoprotein granule	Nucleus, Cytoplasm	1. P-bodies modulation of mRNA metabolism plays a critical factor in the development and progression of cancer{Nsengimana, 2022 #225} 2. Stress granules assembly is increased in tumor{Shi, 2019 #226}
YTHDF3	P-body; cytoplasmic stress granule; neuronal ribonucleoprotein granule	Cytoplasm	1. P-bodies modulation of mRNA metabolism plays a critical factor in the development and progression of cancer{Nsengimana, 2022 #225} 2. Stress granules assembly is increased in tumor{Shi, 2019 #226}
TAF15	Nuclear protein granule	Nucleus, Cytoplasm	The ability of oncogenic transformation in relevant cancers can be influenced by aberrant gene transcription occurring through loci-specific phase separation{Thandapani, 2019 #227}
OCT-4	MED1 droplets at SEs	Nucleus and Cytoplasm	SEs mediate transcriptional addiction in diverse cancers{Qiao, 2016 #228}
YAP	YAP-TEAD complex/ YAP-TAZ-TEAD complex	Nucleus and Cytoplasm	The compound is over-hyperactivated, and also confers a great oncogenic activity in cancer{Qiao, 2016 #228}
TAZ	YAP-TAZ-TEAD complex	Nucleus and Cytoplasm	The compound is over-hyperactivated, and also confers a great oncogenic activity in cancer{Qiao, 2016 #228}.
DDX3	Cytoplasmic stress granule	Nucleus, cytoplasm and plasma membrane	Cancer-associated mutations of DDX3X cause SG hyper-assembly and translation impairment{Valentin-Vega, 2016 #229}
HSF1	HSF1 nuclear stress bodies	Nucleus	HSF1 foci are preferentially located in cancer cells of primary human{Gaglia, 2020 #230}v

### 4.3 LLPS in immune signaling pathways

However, the literatures associated with LLPS and immune signaling progress was scarce in cancer, and there is a paucity of bibliometric research on these topics. Therefore, based on the relevant research background, VOSviewer software, and CiteSpace software visualization, we found the number of enrolled literatures on this topic remains low, with nearly 57 documents published from 1992-2024. Furthermore, with an average citation rate of ≥ 10 per year, only 8 high-quality studies were identified, indicating the correlated research between LLPS and immune signaling response is still in the infant stage. Meanwhile, we also searched relevant literatures in the field of bone tumor, according to the above mentioned method. The outcome was none.

### 4.4 LLPS and immune signaling pathways in OS disease

On 15 February, 2024, the journal Lancet Child & Adolescent Health onlined “Holistic support for children with rare disease”. The author, Siyuan Li, a Ph.D. student engaged in the regenerative medicine of biomedical engineering, was also a patient with OS disease in childhood (97). “The former patient has become a doctoral student in biomedical engineering, and her crutches have not only seen her on the road to recovery but also given her a guiding light to a new field,” as reviewed by the Lancet Child & Adolescent Health journal. It is very lucky for children with OS to be like Siyuan Li. Therefore, increased efforts are necessary to explore the pathogenesis that could be utilized in preclinical research and clinical trials, especially the immunotherapy with LLPS in OS.

### 4.5 LLPS in other aspects of tumor immunology, tumor biology and chronic inflammation

LLPS could potentially aid in immune evasion by promoting the creation of biomolecular condensates that trap immune signaling proteins or regulate the production of immune checkpoint molecules. This means that cancer cells could exploit LLPS to gather proteins that hinder the stimulation of immune cells, ultimately establishing a microenvironment that is less detectable by the immune system (98). The spatial arrangement of signaling molecules mediated by LLPS can also influence the recruitment and activation of immune cells, shaping immune cell communication. This organization may facilitate the formation of signaling complexes at the immune synapse, critical for immune cell activation. Conversely, it could also cause the segregation of signaling molecules away from the synapse, potentially dampening the immune response (99).

Additionally, LLPS could potentially contribute to the development of an immunosuppressive tumor microenvironment (TME) by influencing the positioning and behavior of immune cells in the tumor. This process could result in the creation of condensates that draw in immunosuppressive cells such as Tregs or MDSCs, which have the ability to dampen the function of cytotoxic T cells. Moreover, LLPS might impact the release of immunosuppressive cytokines, leading to a TME that is not conducive to successful immune attacks on cancer cells (100). Overall, the emerging role of LLPS in the tumor microenvironment highlights its critical influence on cancer progression, immune evasion, and therapeutic resistance. Understanding the mechanisms by which LLPS modulates oncogenic signaling and immune responses not only provides new insights into cancer biology but also offers promising avenues for developing novel therapeutic strategies targeting LLPS-related pathways.

Meanwhile, the study of LLPS in the field of tumor associated macrophages (TAMs) is an emerging area of research that has shown promising implications in both tumor progression and immune responses. In TAMs, LLPS might regulate signal transduction processes, ultimately leading to an upregulation of immune-suppressive molecules, such as PD-L1. It might also be involved in controlling the secretion of cytokines like IL-10 and TGF-β, and in regulating the metabolism of TAMs. Furthermore, LLPS could potentially influence the phagocytic function of TAMs by modifying the aggregation state of proteins associated with intracellular endocytosis, which in turn affects TAMs’ ability to engulf tumor cells (101, 102).

At last, chronic inflammation is also greatly influenced by LLPS, which affects intracellular signaling pathways, modulates molecular dynamics during inflammatory responses, and determines the polarization state of immune cells (103). LLPS can also affect the functionality of macrophages and potentially play a role in the formation of inflammasomes (104, 105).

### 4.6 Immunotherapy with LLPS

Recently, the landscape of tumor treatment has shifted toward tumor immunotherapy, which stands as a beacon in research and therapy, and is now widely recognized as the fourth line of treatment (106). Tumor immunotherapy encompasses immune checkpoint blockades (ICBs) and chimeric antigen receptor T cell (CAR-T) immunotherapy. Broadly speaking, LLPS could impact tumorigenesis through various signaling pathways, it is essential to devise practical strategies for treating these cancer-associated proteins. For instance, we can disrupt the LLPS process, target cancer drugs within bio-molecular condensates, and modify LLPS by interfering with PTMs (posttranslational modifications, RTMs).

For example, a study conducted in 2024 showed that Svg3, a nature-inspired oligonucleotide, is a potent cGAS agonist that activates cGAS-STING in tumor immunotherapy. The hairpin-shaped Svg3 exhibited strong binding to cGAS and facilitated LLPS to generate Svg3-cGAS liquid-like condensates, which led to specific activation of cGAS and robust IFN-1 responses. Thrillingly, Svg3 surpasses several cutting-edge STING agonists in human and murine cells/tissues (107). Si Sun, et al. (108) have found that high levels of CAL protein-coding gene transcription were significantly associated with poor prognosis in KIRP and were also linked to specific targeted therapies. The inhibitor of LLPS could also increase the effectiveness of paclitaxel and cisplatin in killing cancer cells. Targeting CAL signatures might be a promising therapeutic approach with LLPS modulating synergy. Merlin (NF2/schwannoma), is a tumor suppressor protein and boots innate immunity against cancer (30, 109). While, Merlin can also be found in various malignancies with genetic inactivation and mutations, such as skin cancer, type 2 neurofibromatosis, schwannomas, and colorectal cancer (110). A recent study by Meng et al. revealed that by forming LLPS condensates with IRF3, the mutant FERM domain of Merlin further obstructed the anticancer immunity signaling pathway. Based on the above studies, in NF2-related cancer, trying to prevent the formation of intracellular membranes structures of NF2 could restore the antitumor immune responses mediated by the cGAS-STING pathway (30).

Even though all of these initiatives are still in their early stages, we anticipate that delving into the study of LLPS will lead to a more profound comprehension of pathological processes in cancer and reveal fresh possibilities for treatment.

### 4.7 Unsolved outstanding problems

While the field is still in its infancy, further investigations are needed to thoroughly examine the functions of phase separation in immunity, including advancements in technology and physiological research. Looking ahead, we predict that the following areas will present exciting avenues for exploration.

#### 4.7.1 Detecting techniques

Apprehending the LLPS condensates organization internally will greatly benefit the design of antagonists and agonists by disrupting liquid-like condensates and the involved immune signaling responses. While crystallography and electron microscopy have been employed to ascertain the internal structure and arrangement of the condensates, the application for liquid-like objects remains restricted. Since LLPS occurs widely in a 4D environment, Breakthroughs in nuclear magnetic resonance imaging, computational simulations, spatial omics technology, and the invention of new fluorescent probes might open up new possibilities for approaching this issue, which could be pivotal for immunotherapy with LLPS, and even for implementing personalized therapy in clinical settings (111).

#### 4.7.2 LLPS for the immunological synapse

Up to now, the majority of LLPS research has mainly focused on a single situation, just like the plasma membrane (TCR, BCR) or the cytoplasm (cGAS, RIG-1). Nonetheless, studies between LLPS and immunological synapse are still limited, which consist of five environments: the immune cell’s cytosol and plasma membrane, along with the intermembrane space, and the antigen-presenting cell’s plasma membrane and cytosol. The interaction between these diverse environments is facilitated by numerous ligand-receptor pairs, which can affect their assembly structures and facilitate two-way signaling. By utilizing a multiple-membrane reconstitution system alongside light-sheet microscopy on live-cell conjugates, a comprehensive understanding of the phase separation behavior at immunological synapses can be achieved.

### 4.8 Advantages and limitations

#### 4.8.1 Advantages

In this research, we performed an integrated analysis of the relationship between immune and LLPS biological processes in cancer for the first time, using bibliometric estimation to present the research status, key areas of focus, and potential future research trends in this area. Moreover, by utilizing R package “bibliometrix” software, CiteSpace, and VOSviewer analyses, we have ensured the accuracy and reliability of the data, allowing us to deeply and thoroughly elucidate the evolving trends in the pathogenic mechanisms and immunotherapy associated with LLPS. We also further assessed the state of the research at the intersection of LLPS, immune signaling responses, and bone tumor disease. More surprisingly, no literature was found. Nevertheless, we cannot deny the significant importance of LLPS in immunity, especially in tumor disease. In the subcellular section, scientific research on LLPS has uncovered intracellular compartments or new membrane-less organelles involved in signaling transduction. In the molecular section, LLPS highlights the significance of unstructured protein domains and weak interactions. These components, often overlooked in previous studies on protein-protein interactions, play a crucial role in driving LLPS. In the physiological section, LLPS provides pioneering insights into the mechanisms of cellular decision-making processes in immune signaling responses.

#### 4.8.2 Limitations

However, the present study also has some inherent limitations. First, the documents were only obtained from the WoS database, not from Scopus or Embase, which may potentially lead to biased results. Future research may need to search additional databases or other bibliometric tools to verify these findings. Meanwhile, as the field of LLPS in immune signaling response is still an emerging research area, related literatures, especially those focusing on cancer, are scarce. Additionally, new online publications in reputable journals may have been overlooked due to their lower citation counts. The lack of relevant keywords with strong citation bursts in the CiteSpace software indicates a scarcity of literature on this topic. Furhtermore, the use of different parameters in the CiteSpace software may have impacted the output data, leading to slight variations in the results.

## 5 Conclusions

In conclusion, this study is the first to conduct a bibliometric analysis that scientifically and comprehensively examines the correlation between liquid-liquid phase separation (LLPS) and the immune signaling system in cancer research trends over the past 30 years. It has systematically summarized global publication trends and helped researchers identify key authors, institutions, and journals in this field. Additionally, a qualitative analysis has also been conducted. Despite this research area being in its early stages, illustrating the interconnected structures and communications between cancer and immune signaling with LLPS within a spatial framework will provide deeper insights into the molecular mechanisms of cancer development and enhance the effectiveness of current immunotherapies. At the same time, the development of compounds that target LLPS and the utilization of LLPS as a biomarker for cancer diagnosis and prognosis are also key points that scientists need to focus on. Nevertheless, the intricate nature of LLPS, the constantly changing condensates, and the requirement for specificity present challenges that need to be addressed thoughtfully.

## Data Availability

The datasets presented in this study can be found in online repositories. The names of the repository/repositories and accession number(s) can be found in the article/**Supplementary Material**.

## References

[1] Stratton MR Campbell PJ Futreal PA . The cancer genome. Nature. (2009) 458:719–24. doi: 10.1038/nature07943 PMC2821689 19360079

[2] Podlaha O Riester M De S Michor F . Evolution of the cancer genome. Trends Genet. (2012) 28:155–63. doi: 10.1016/j.tig.2012.01.003 PMC3711268 22342180

[3] Matsushita H Vesely MD Koboldt DC Rickert CG Uppaluri R Magrini VJ . Cancer exome analysis reveals a T-cell-dependent mechanism of cancer immunoediting. Nature. (2012) 482:400–4. doi: 10.1038/nature10755 PMC3874809 22318521

[4] Schreiber RD Old LJ Smyth MJ . Cancer immunoediting: integrating immunity's roles in cancer suppression and promotion. Science. (2011) 331:1565–70. doi: 10.1126/science.1203486 21436444

[5] Vesely MD Kershaw MH Schreiber RD Smyth MJ . Natural innate and adaptive immunity to cancer. Annu Rev Immunol. (2011) 29:235–71. doi: 10.1146/annurev-immunol-031210-101324 21219185

[6] Grivennikov SI Greten FR Karin M . Immunity. Inflammation Cancer Cell. (2010) 140(6):883–99. doi: 10.1016/j.cell.2010.01.025 PMC2866629 20303878

[7] Seager RJ Hajal C Spill F Kamm RD Zaman MH . Dynamic interplay between tumour, stroma and immune system can drive or prevent tumour progression. Converg Sci Phys Oncol (2017) 3:034002. doi: 10.1088/2057-1739/aa7e86 30079253 PMC6070160

[8] Demaria O Cornen S Daeron M Morel Y Medzhitov R Vivier E . Harnessing innate immunity in cancer therapy. Nature. (2019) 574:45–56. doi: 10.1038/s41586-019-1593-5 31578484

[9] Woo SR Corrales L Gajewski TF . Innate immune recognition of cancer. Annu Rev Immunol. (2015) 33:445–74. doi: 10.1146/annurev-immunol-032414-112043 25622193

[10] Corrales L Matson V Flood B Spranger S Gajewski TF . Innate immune signaling and regulation in cancer immunotherapy. Cell Res. (2017) 27:96–108. doi: 10.1038/cr.2016.149 27981969 PMC5223230

[11] Alderton GK Bordon Y . Tumour immunotherapy–leukocytes take up the fight. Nat Rev Immunol. (2012) 12:237. doi: 10.1038/nri3197 22545289

[12] Yuen GJ Demissie E Pillai S . B lymphocytes and cancer: a love-hate relationship. Trends Cancer. (2016) 2:747–57. doi: 10.1016/j.trecan.2016.10.010 PMC5472356 28626801

[13] Khong HT Restifo NP . Natural selection of tumor variants in the generation of "tumor escape" phenotypes. Nat Immunol. (2002) 3:999–1005. doi: 10.1038/ni1102-999 12407407 PMC1508168

[14] Thomas DA Massague J . TGF-beta directly targets cytotoxic T cell functions during tumor evasion of immune surveillance. Cancer Cell. (2005) 8:369–80. doi: 10.1016/j.ccr.2005.10.012 16286245

[15] Blank C Gajewski TF Mackensen A . Interaction of PD-L1 on tumor cells with PD-1 on tumor-specific T cells as a mechanism of immune evasion: implications for tumor immunotherapy. Cancer Immunol Immunother. (2005) 54:307–14. doi: 10.1007/s00262-004-0593-x PMC11032914 15599732

[16] Drake CG Jaffee E Pardoll DM . Mechanisms of immune evasion by tumors. Adv Immunol. (2006) 90:51–81. doi: 10.1016/S0065-2776(06)90002-9 16730261

[17] Rabinovich GA Gabrilovich D Sotomayor EM . Immunosuppressive strategies that are mediated by tumor cells. Annu Rev Immunol. (2007) 25:267–96. doi: 10.1146/annurev.immunol.25.022106.141609 PMC2895922 17134371

[18] Hodi FS O'Day SJ McDermott DF Weber RW Sosman JA Haanen JB . Improved survival with ipilimumab in patients with metastatic melanoma. N Engl J Med. (2010) 363:711–23. doi: 10.1056/NEJMoa1003466 PMC3549297 20525992

[19] Motzer RJ Escudier B McDermott DF George S Hammers HJ Srinivas S . Nivolumab versus everolimus in advanced renal-cell carcinoma. N Engl J Med. (2015) 373:1803–13. doi: 10.1056/NEJMoa1510665 PMC5719487 26406148

[20] Ribas A . Releasing the brakes on cancer immunotherapy. N Engl J Med. (2015) 373:1490–2. doi: 10.1056/NEJMp1510079 26348216

[21] Schmid P Adams S Rugo HS Schneeweiss A Barrios CH Iwata H . Atezolizumab and nab-paclitaxel in advanced triple-negative breast cancer. N Engl J Med. (2018) 379:2108–21. doi: 10.1056/NEJMoa1809615 30345906

[22] Gandhi L Rodriguez-Abreu D Gadgeel S Esteban E Felip E De Angelis F . Pembrolizumab plus chemotherapy in metastatic non-small-cell lung cancer. N Engl J Med. (2018) 378:2078–92. doi: 10.1056/NEJMoa1801005 29658856

[23] Forde PM Chaft JE Pardoll DM . Neoadjuvant PD-1 blockade in resectable lung cancer. N Engl J Med. (2018) 379:e14. doi: 10.1056/NEJMoa1716078 30157404

[24] Wei SC Duffy CR Allison JP . Fundamental mechanisms of immune checkpoint blockade therapy. Cancer Discovery. (2018) 8:1069–86. doi: 10.1158/2159-8290.CD-18-0367 30115704

[25] Alberti S Gladfelter A Mittag T . Considerations and challenges in studying liquid-liquid phase separation and biomolecular condensates. Cell. (2019) 176:419–34. doi: 10.1016/j.cell.2018.12.035 PMC6445271 30682370

[26] Che X Wu J Liu H Su J Chen X . Cellular liquid-liquid phase separation: Concept. functions regulations detections J Cell Physiol. (2023) 238:847–65. doi: 10.1002/jcp.30980 36870067

[27] Zhang JZ Mehta S Zhang J . Liquid-liquid phase separation: a principal organizer of the cell's biochemical activity architecture. Trends Pharmacol Sci. (2021) 42:845–56. doi: 10.1016/j.tips.2021.07.003 PMC8858030 34373114

[28] Peng Q Tan S Xia L Wu N Oyang L Tang Y . Phase separation in Cancer: From the Impacts and Mechanisms to Treatment potentials. Int J Biol Sci. (2022) 18:5103–22. doi: 10.7150/ijbs.75410 PMC9379413 35982902

[29] Alberti S Dormann D . Liquid-liquid phase separation in disease. Annu Rev Genet. (2019) 53:171–94. doi: 10.1146/annurev-genet-112618-043527 31430179

[30] Meng F Yu Z Zhang D Chen S Guan H Zhou R . Induced phase separation of mutant NF2 imprisons the cGAS-STING machinery to abrogate antitumor immunity. Mol Cell. (2021) 81:4147–4164 e7. doi: 10.1016/j.molcel.2021.07.040 34453890

[31] Dine E Gil AA Uribe G Brangwynne CP Toettcher JE . Protein phase separation provides long-term memory of transient spatial stimuli. Cell Syst. (2018) 6:655–663 e5. doi: 10.1016/j.cels.2018.05.002 29859829 PMC6023754

[32] Nielsen MW Andersen JP Schiebinger L Schneider JW . One and a half million medical literatures reveal a link between author gender and attention to gender and sex analysis. Nat Hum Behav. (2017) 1:791–6. doi: 10.1038/s41562-017-0235-x 31024130

[33] Boudry C Baudouin C Mouriaux F . International publication trends in dry eye disease research: A bibliometric analysis. Ocul Surf. (2018) 16:173–9. doi: 10.1016/j.jtos.2017.10.002 29031646

[34] Fu HZ Wang MH Ho YS . Mapping of drinking water research: a bibliometric analysis of research output during 1992-2011. Sci Total Environ. (2013) 443:757–65. doi: 10.1016/j.scitotenv.2012.11.061 23228721

[35] Wu H Li Y Tong L Wang Y Sun Z . Worldwide research tendency and hotspots on hip fracture: a 20-year bibliometric analysis. Arch Osteoporos. (2021) 16:73. doi: 10.1007/s11657-021-00929-2 33866438

[36] Liao Z Wei W Yang M Kuang X Shi J . Academic publication of neurodegenerative diseases from a bibliographic perspective: A comparative scientometric analysis. Front Aging Neurosci. (2021) 13:722944. doi: 10.3389/fnagi.2021.722944 34803653 PMC8601281

[37] Yan P Li M Li J Lu Z Hui X Bai Y . Bibliometric analysis and systematic review of global coronavirus research trends before COVID-19: prospects and implications for COVID-19 research. Front Med (Lausanne). (2021) 8:729138. doi: 10.3389/fmed.2021.729138 34869424 PMC8635101

[38] Liu T Yang L Mao H Ma F Wang Y Zhan Y . Knowledge domain and emerging trends in podocyte injury research from 1994 to 2021: A bibliometric and visualized analysis. Front Pharmacol. (2021) 12:772386. doi: 10.3389/fphar.2021.772386 34925030 PMC8678497

[39] Xing D Zhao Y Dong S Lin J . Global research trends in stem cells for osteoarthritis: a bibliometric and visualized study. Int J Rheum Dis. (2018) 21:1372–84. doi: 10.1111/apl.2018.21.issue-7 29968331

[40] Xiong W Wang S Wei Z Cai Y Li B Lin F . Knowledge domain and hotspots predict concerning electroactive biomaterials applied in tissue engineering: A bibliometric and visualized analysis from 2011 to 2021. Front Bioeng Biotechnol. (2022) 10:904629. doi: 10.3389/fbioe.2022.904629 35677303 PMC9168279

[41] Case LB Ditlev JA Rosen MK . Regulation of transmembrane signaling by phase separation. Annu Rev Biophys. (2019) 48:465–94. doi: 10.1146/annurev-biophys-052118-115534 PMC6771929 30951647

[42] Dustin ML Groves JT . Receptor signaling clusters in the immune synapse. Annu Rev Biophys. (2012) 41:543–56. doi: 10.1146/annurev-biophys-042910-155238 PMC4000727 22404679

[43] Jaqaman K Ditlev JA . Biomolecular condensates in membrane receptor signaling. Curr Opin Cell Biol. (2021) 69:48–54. doi: 10.1016/j.ceb.2020.12.006 33461072 PMC8058224

[44] Liu B Chen W Evavold BD Zhu C . Accumulation of dynamic catch bonds between TCR and agonist peptide-MHC triggers T cell signaling. Cell. (2014) 157:357–68. doi: 10.1016/j.cell.2014.02.053 PMC4123688 24725404

[45] Nishi H Furuhashi K Cullere X Saggu G Miller MJ Chen Y . Neutrophil FcgammaRIIA promotes IgG-mediated glomerular neutrophil capture via Abl/Src kinases. J Clin Invest. (2017) 127:3810–26. doi: 10.1172/JCI94039 PMC5617671 28891817

[46] Wan Z Chen X Chen H Ji Q Chen Y Wang J . The activation of IgM- or isotype-switched IgG- and IgE-BCR exhibits distinct mechanical force sensitivity and threshold. Elife. (2015) 4:e06925. doi: 10.7554/eLife.06925 26258882 PMC4555871

[47] Bunnell SC Hong DI Kardon JR Yamazaki T McGlade CJ Barr VA . T cell receptor ligation induces the formation of dynamically regulated signaling assemblies. J Cell Biol. (2002) 158:1263–75. doi: 10.1083/jcb.200203043 PMC2173229 12356870

[48] Hui E Cheung J Zhu J Su X Taylor MJ Wallweber HA . T cell costimulatory receptor CD28 is a primary target for PD-1-mediated inhibition. Science. (2017) 355:1428–33. doi: 10.1126/science.aaf1292 PMC6286077 28280247

[49] Barda-Saad M Braiman A Titerence R Bunnell SC Barr VA Samelson LE . Dynamic molecular interactions linking the T cell antigen receptor to the actin cytoskeleton. Nat Immunol. (2005) 6:80–9. doi: 10.1038/ni1143 15558067

[50] Houtman JC Yamaguchi H Barda-Saad M Braiman A Bowden B Appella E . Oligomerization of signaling complexes by the multipoint binding of GRB2 to both LAT and SOS1. Nat Struct Mol Biol. (2006) 13:798–805. doi: 10.1038/nsmb1133 16906159

[51] Su X Ditlev JA Hui E Xing W Banjade S Okrut J . Phase separation of signaling molecules promotes T cell receptor signal transduction. Science. (2016) 352:595–9. doi: 10.1126/science.aad9964 PMC4892427 27056844

[52] Kortum RL Balagopalan L Alexander CP Garcia J Pinski JM Merrill RK . The ability of Sos1 to oligomerize the adaptor protein LAT is separable from its guanine nucleotide exchange activity *in vivo* . Sci Signal. (2013) 6:ra99. doi: 10.1126/scisignal.2004494 24222714 PMC4259567

[53] Zeng L Palaia I Saric A Su X . PLCgamma1 promotes phase separation of T cell signaling components. J Cell Biol. (2021) 220(6). doi: 10.1083/jcb.202009154 PMC8094118 33929486

[54] Pielak RM O'Donoghue GP Lin JJ Alfieri KN Fay NC Low-Nam ST . Early T cell receptor signals globally modulate ligand:receptor affinities during antigen discrimination. Proc Natl Acad Sci U.S.A. (2017) 114:12190–5. doi: 10.1073/pnas.1613140114 PMC5699024 29087297

[55] Lin JJY Low-Nam ST Alfieri KN McAffee DB Fay NC Groves JT . Mapping the stochastic sequence of individual ligand-receptor binding events to cellular activation: T cells act on the rare events. Sci Signal. (2019) 12. doi: 10.1126/scisignal.aat8715 PMC6598675 30647147

[56] Xiao Q McAtee CK Su X . Phase separation in immune signalling. Nat Rev Immunol. (2022) 22:188–99. doi: 10.1038/s41577-021-00572-5 PMC9674404 34230650

[57] Wong LE Bhatt A Erdmann PS Hou Z Maier J Pirkuliyeva S . Tripartite phase separation of two signal effectors with vesicles priming B cell responsiveness. Nat Commun. (2020) 11:848. doi: 10.1038/s41467-020-14544-1 32051419 PMC7016142

[58] Engelke M Pirkuliyeva S Kuhn J Wong L Boyken J Herrmann N . Macromolecular assembly of the adaptor SLP-65 at intracellular vesicles in resting B cells. Sci Signal. (2014) 7:ra79. doi: 10.1126/scitranslmed.2005104 25140054

[59] Oellerich T Bremes V Neumann K Bohnenberger H Dittmann K Hsiao HH . The B-cell antigen receptor signals through a preformed transducer module of SLP65 and CIN85. EMBO J. (2011) 30:3620–34. doi: 10.1038/emboj.2011.251 PMC3181483 21822214

[60] Kee BL . It's a phase that EBF1 is going through. Immunity. (2020) 53:1123–5. doi: 10.1016/j.immuni.2020.11.013 33326760

[61] Williamson AP Vale RD . Spatial control of Draper receptor signaling initiates apoptotic cell engulfment. J Cell Biol. (2018) 217:3977–92. doi: 10.1083/jcb.201711175 PMC6219719 30139739

[62] Goodridge HS Reyes CN Becker CA Katsumoto TR Ma J Wolf AJ . Activation of the innate immune receptor Dectin-1 upon formation of a 'phagocytic synapse'. Nature. (2011) 472:471–5. doi: 10.1038/nature10071 PMC3084546 21525931

[63] Sun L Wu J Du F Chen X Chen ZJ . Cyclic GMP-AMP synthase is a cytosolic DNA sensor that activates the type I interferon pathway. Science. (2013) 339:786–91. doi: 10.1126/science.1232458 PMC3863629 23258413

[64] Wu J Sun L Chen X Du F Shi H Chen C . Cyclic GMP-AMP is an endogenous second messenger in innate immune signaling by cytosolic DNA. Science. (2013) 339:826–30. doi: 10.1126/science.1229963 PMC3855410 23258412

[65] Du M Chen ZJ . DNA-induced liquid phase condensation of cGAS activates innate immune signaling. Science. (2018) 361:704–9. doi: 10.1126/science.aat1022 PMC9417938 29976794

[66] Zhang W Liu W Jia L Chen D Chang I Lake M . Targeting KDM4A epigenetically activates tumor-cell-intrinsic immunity by inducing DNA replication stress. Mol Cell. (2021) 81:2148–2165 e9. doi: 10.1016/j.molcel.2021.02.038 33743195 PMC8141018

[67] Lin H Jiang M Liu L Yang Z Ma Z Liu S . The long noncoding RNA Lnczc3h7a promotes a TRIM25-mediated RIG-I antiviral innate immune response. Nat Immunol. (2019) 20:812–23. doi: 10.1038/s41590-019-0379-0 31036902

[68] Gack MU Shin YC Joo CH Urano T Liang C Sun L . TRIM25 RING-finger E3 ubiquitin ligase is essential for RIG-I-mediated antiviral activity. Nature. (2007) 446:916–20. doi: 10.1038/nature05732 17392790

[69] Arimoto K Takahashi H Hishiki T Konishi H Fujita T Shimotohno K . Negative regulation of the RIG-I signaling by the ubiquitin ligase RNF125. Proc Natl Acad Sci U.S.A. (2007) 104:7500–5. doi: 10.1073/pnas.0611551104 PMC1863485 17460044

[70] Jobe F Simpson J Hawes P Guzman E Bailey D . Respiratory syncytial virus sequesters NF-kappaB subunit p65 to cytoplasmic inclusion bodies to inhibit innate immune signaling. J Virol. (2020) 94. doi: 10.1128/JVI.01380-20 PMC7592213 32878896

[71] Qin Z Fang X Sun W Ma Z Dai T Wang S . Deactylation by SIRT1 enables liquid-liquid phase separation of IRF3/IRF7 in innate antiviral immunity. Nat Immunol. (2022) 23:1193–207. doi: 10.1038/s41590-022-01269-0 35879450

[72] Li Y Peng Q Wang L . EphA2 as a phase separation protein associated with ferroptosis and immune cell infiltration in colorectal cancer. Aging (Albany NY). (2023) 15:12952–65. doi: 10.18632/aging.205212 PMC10713424 37980165

[73] Zhang Z Boskovic Z Hussain MM Hu W Inouye C Kim HJ . Chemical perturbation of an intrinsically disordered region of TFIID distinguishes two modes of transcription initiation. Elife. (2015) 4. doi: 10.7554/eLife.07777 PMC4582147 26314865

[74] Ban D Iconaru LI Ramanathan A Zuo J Kriwacki RW . A small molecule causes a population shift in the conformational landscape of an intrinsically disordered protein. J Am Chem Soc. (2017) 139:13692–700. doi: 10.1021/jacs.7b01380 PMC5962290 28885015

[75] Iconaru LI Ban D Bharatham K Ramanathan A Zhang W Shelat AA . Discovery of small molecules that inhibit the disordered protein, p27(Kip1). Sci Rep. (2015) 5:15686. doi: 10.1038/srep15686 26507530 PMC4623604

[76] Zhang H Ji X Li P Liu C Lou J Wang Z . Liquid-liquid phase separation in biology: mechanisms, physiological functions and human diseases. Sci China Life Sci. (2020) 63:953–85. doi: 10.1007/s11427-020-1702-x 32548680

[77] Li P Banjade S Cheng HC Kim S Chen B Guo L . Phase transitions in the assembly of multivalent signalling proteins. Nature. (2012) 483:336–40. doi: 10.1038/nature10879 PMC3343696 22398450

[78] Iakoucheva LM Radivojac P Brown CJ O'Connor TR Sikes JG Obradovic Z . The importance of intrinsic disorder for protein phosphorylation. Nucleic Acids Res. (2004) 32:1037–49. doi: 10.1093/nar/gkh253 PMC373391 14960716

[79] Peng PH Hsu KW Wu KJ . Liquid-liquid phase separation (LLPS) in cellular physiology and tumor biology. Am J Cancer Res. (2021) 11:3766–76. PMC8414392 34522448

[80] Spegg V Altmeyer M . Biomolecular condensates at sites of DNA damage: More than just a phase. DNA Repair (Amst). (2021) 106:103179. doi: 10.1016/j.dnarep.2021.103179 34311273 PMC7612016

[81] Dao TP Castaneda CA . Ubiquitin-modulated phase separation of shuttle proteins: does condensate formation promote protein degradation? Bioessays. (2020) 42:e2000036. doi: 10.1002/bies.202000036 32881044 PMC7737676

[82] Hnisz D Shrinivas K Young RA Chakraborty AK Sharp PA . A phase separation model for transcriptional control. Cell. (2017) 169(1):13–23. doi: 10.1016/j.cell.2017.02.007 28340338 PMC5432200

[83] Patel A Lee HO Jawerth L Maharana S Jahnel M Hein MY . A liquid-to-solid phase transition of the ALS protein FUS accelerated by disease mutation. Cell. (2015) 162:1066–77. doi: 10.1016/j.cell.2015.07.047 26317470

[84] Maharana S Wang J Papadopoulos DK Richter D Pozniakovsky A Poser I . RNA buffers the phase separation behavior of prion-like RNA binding proteins. Science. (2018) 360:918–21. doi: 10.1126/science.aar7366 PMC6091854 29650702

[85] Mann JR Gleixner AM Mauna JC Gomes E DeChellis-Marks MR Needham PG . RNA binding antagonizes neurotoxic phase transitions of TDP-43. Neuron. (2019) 102:321–338 e8. doi: 10.1016/j.neuron.2019.01.048 30826182 PMC6472983

[86] Gasset-Rosa F Lu S Yu H Chen C Melamed Z Guo L . Cytoplasmic TDP-43 de-mixing independent of stress granules drives inhibition of nuclear import, loss of nuclear TDP-43, and cell death. Neuron. (2019) 102:339–357 e7. doi: 10.1016/j.neuron.2019.02.038 30853299 PMC6548321

[87] Lin Y Protter DS Rosen MK Parker R . Formation and maturation of phase-separated liquid droplets by RNA-binding proteins. Mol Cell. (2015) 60:208–19. doi: 10.1016/j.molcel.2015.08.018 PMC4609299 26412307

[88] Nott TJ Petsalaki E Farber P Jervis D Fussner E Plochowietz A . Phase transition of a disordered nuage protein generates environmentally responsive membraneless organelles. Mol Cell. (2015) 57:936–47. doi: 10.1016/j.molcel.2015.01.013 PMC4352761 25747659

[89] Zhang X Vigers M McCarty J Rauch JN Fredrickson GH Wilson MZ . The proline-rich domain promotes Tau liquid-liquid phase separation in cells. J Cell Biol. (2020) 219. doi: 10.1083/jcb.202006054 PMC7594490 32997736

[90] Zhang X Lin Y Eschmann NA Zhou H Rauch JN Hernandez I . RNA stores tau reversibly in complex coacervates. PloS Biol. (2017) 15:e2002183. doi: 10.1371/journal.pbio.2002183 28683104 PMC5500003

[91] Yamazaki T Souquere S Chujo T Kobelke S Chong YS Fox AH . Functional Domains of NEAT1 Architectural lncRNA Induce Paraspeckle Assembly through Phase Separation. Mol Cell. (2018) 70:1038–1053 e7. doi: 10.1016/j.molcel.2018.05.019 29932899

[92] Plys AJ Davis CP Kim J Rizki G Keenen MM Marr SK . Phase separation of Polycomb-repressive complex 1 is governed by a charged disordered region of CBX2. Genes Dev. (2019) 33:799–813. doi: 10.1101/gad.326488.119 31171700 PMC6601514

[93] Kilic S Lezaja A Gatti M Bianco E Michelena J Imhof R . Phase separation of 53BP1 determines liquid-like behavior of DNA repair compartments. EMBO J. (2019) 38:e101379. doi: 10.15252/embj.2018101379 31267591 PMC6694294

[94] de The H Pandolfi PP Chen Z . Acute promyelocytic leukemia: A paradigm for oncoprotein-targeted cure. Cancer Cell. (2017) 32:552–60. doi: 10.1016/j.ccell.2017.10.002 29136503

[95] Cai D Feliciano D Dong P Flores E Gruebele M Porat-Shliom N . Phase separation of YAP reorganizes genome topology for long-term YAP target gene expression. Nat Cell Biol. (2019) 21:1578–89. doi: 10.1038/s41556-019-0433-z PMC8259329 31792379

[96] Bouchard JJ Otero JH Scott DC Szulc E Martin EW Sabri N . Cancer mutations of the tumor suppressor SPOP disrupt the formation of active. Phase-Separated Compartments Mol Cell. (2018) 72:19–36 e8. doi: 10.1016/j.molcel.2018.08.027 30244836 PMC6179159

[97] Li S Hua Y . Holistic support for children with rare disease. Lancet Child Adolesc Health. (2024) 8(6):397. doi: 10.1016/S2352-4642(24)00045-2

[98] Ahn JH Davis ES Daugird TA Zhao S Quiroga IY Uryu H . Phase separation drives aberrant chromatin looping and cancer development. Nature. (2021) 595:591–5. doi: 10.1038/s41586-021-03662-5 PMC8647409 34163069

[99] TaNiue K Akimitsu N . Aberrant phase separation and cancer. FEBS J. (2022) 289:17–39. doi: 10.1111/febs.v289.1 33583140

[100] Li X Song J Yi C . Genome-wide mapping of cellular protein-RNA interactions enabled by chemical crosslinking. Genomics Proteomics Bioinf. (2014) 12:72–8. doi: 10.1016/j.gpb.2014.03.001 PMC4411377 24747191

[101] Haynes NM Chadwick TB Parker BS . The complexity of immune evasion mechanisms throughout the metastatic cascade. Nat Immunol. (2024). doi: 10.1038/s41590-024-01960-4 39285252

[102] Pan Y Yu Y Wang X Zhang T . Tumor-associated macrophages in tumor immunity. Front Immunol. (2020) 11:583084. doi: 10.3389/fimmu.2020.583084 33365025 PMC7751482

[103] Wang W Chen J Yu X Lan HY . Signaling mechanisms of SARS-CoV-2 Nucleocapsid protein in viral infection, cell death and inflammation. Int J Biol Sci. (2022) 18:4704–13. doi: 10.7150/ijbs.72663 PMC9305276 35874957

[104] Jia P Li X Wang X Yao L Xu Y Hu Y . ZMYND8 mediated liquid condensates spatiotemporally decommission the latent super-enhancers during macrophage polarization. Nat Commun. (2021) 12:6535. doi: 10.1038/s41467-021-26864-x 34764296 PMC8586003

[105] Sanders DW Kedersha N Lee DSW Strom AR Drake V Riback JA . Competing protein-RNA interaction networks control multiphase intracellular oganization. Cell. (2020) 181:306–324 e28. doi: 10.1016/j.cell.2020.03.050 32302570 PMC7816278

[106] Li H Ding JY Zhang MJ Yu HJ Sun ZJ . Tertiary lymphoid structures and cytokines interconnections: The implication in cancer immunotherapy. Cancer Lett. (2023) 568:216293. doi: 10.1016/j.canlet.2023.216293 37392991

[107] Zhou S Su T Cheng F Cole J Liu X Zhang B . Engineering cGAS-agonistic oligonucleotides as therapeutics for cancer immunotherapy. Mol Ther Nucleic Acids. (2024) 35:102126. doi: 10.1016/j.omtn.2024.102126 38352859 PMC10863322

[108] Sun S Wang W Li G Xiao M Peng M Cai J . Rational therapeutic targets with biomolecular liquid-liquid phase separation regulating synergy: A pan-cancer analysis. PloS One. (2023) 18:e0287574. doi: 10.1371/journal.pone.0287574 37917664 PMC10621828

[109] Stamenkovic I Yu Q . Merlin, a "magic" linker between extracellular cues and intracellular signaling pathways that regulate cell motility, proliferation, and survival. Curr Protein Pept Sci. (2010) 11:471–84. doi: 10.2174/138920310791824011 PMC2946555 20491622

[110] Asthagiri AR Parry DM Butman JA Kim HJ Tsilou ET Zhuang Z . Neurofibromatosis type 2. Lancet. (2009) 373:1974–86. doi: 10.1016/S0140-6736(09)60259-2 PMC4748851 19476995

[111] Nam AS Chaligne R Landau DA . Integrating genetic and non-genetic determinants of cancer evolution by single-cell multi-omics. Nat Rev Genet. (2021) 22:3–18. doi: 10.1038/s41576-020-0265-5 32807900 PMC8450921

