# Correction: Liquid-liquid phase separation drives immune signaling transduction in cancer: a bibliometric and visualized study from 1992 to 2024

**DOI:** 10.3389/fonc.2025.1675771

**Published:** 2025-08-15

**Authors:** Yanhong Pei, Haijie Liang, Yu Guo, Boyang Wang, Han Wu, Zhijian Jin, Shanyi Lin, Fanwei Zeng, Yifan Wu, Qianyu Shi, Jiuhui Xu, Yi Huang, Tingting Ren, Jiarui Liu, Wei Guo

**Affiliations:** ^1^ Department of Bone Tumor, Peking University People’s Hospital, Beijing, China; ^2^ Department of Anatomy, Histology and Embryology, School of Basic Medical Sciences, Health Science Center, Peking University, Beijing, China; ^3^ Neuroscience Research Institute, Peking University, Beijing, China

**Keywords:** liquid liquid phase separation (LLPS), immune transduction, bibliometric, CiteSpace, cancer

An incorrect number was provided for the “National Natural Science Foundation of China”. The correct number is “82072970”.

The original version of this article has been updated.

