# Supplementary figures and images for: Liquid-liquid phase separation drives immune signaling transduction in cancer: a bibliometric and visualized study from 1992 to 2024
Source: Front Oncol. 2025 Mar 4;15:1509457. doi: 10.3389/fonc.2025.1509457 (PMC11913689; doi:10.3389/fonc.2025.1509457)

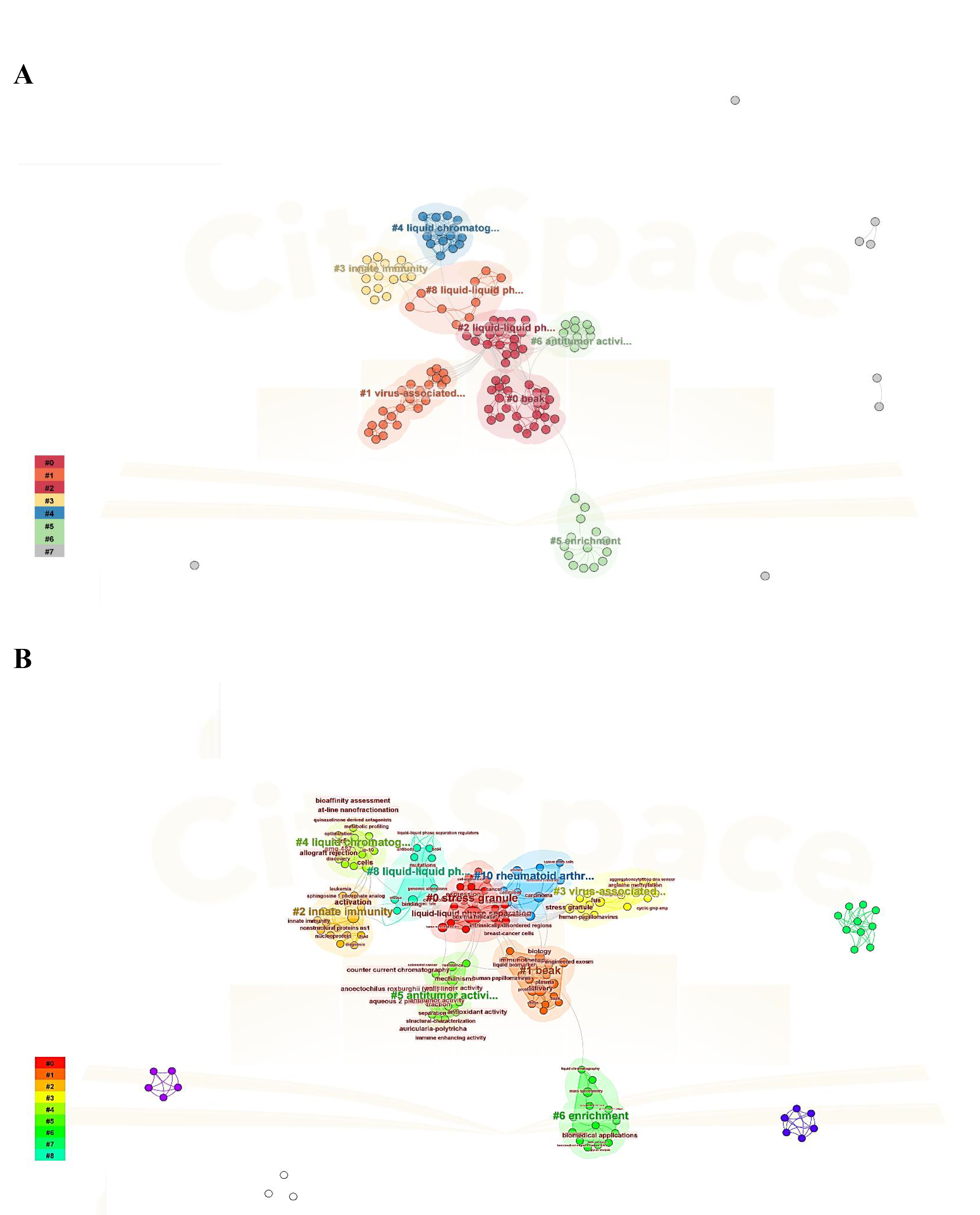

Supplement: Supplementary file 1 [file Image1.tif]

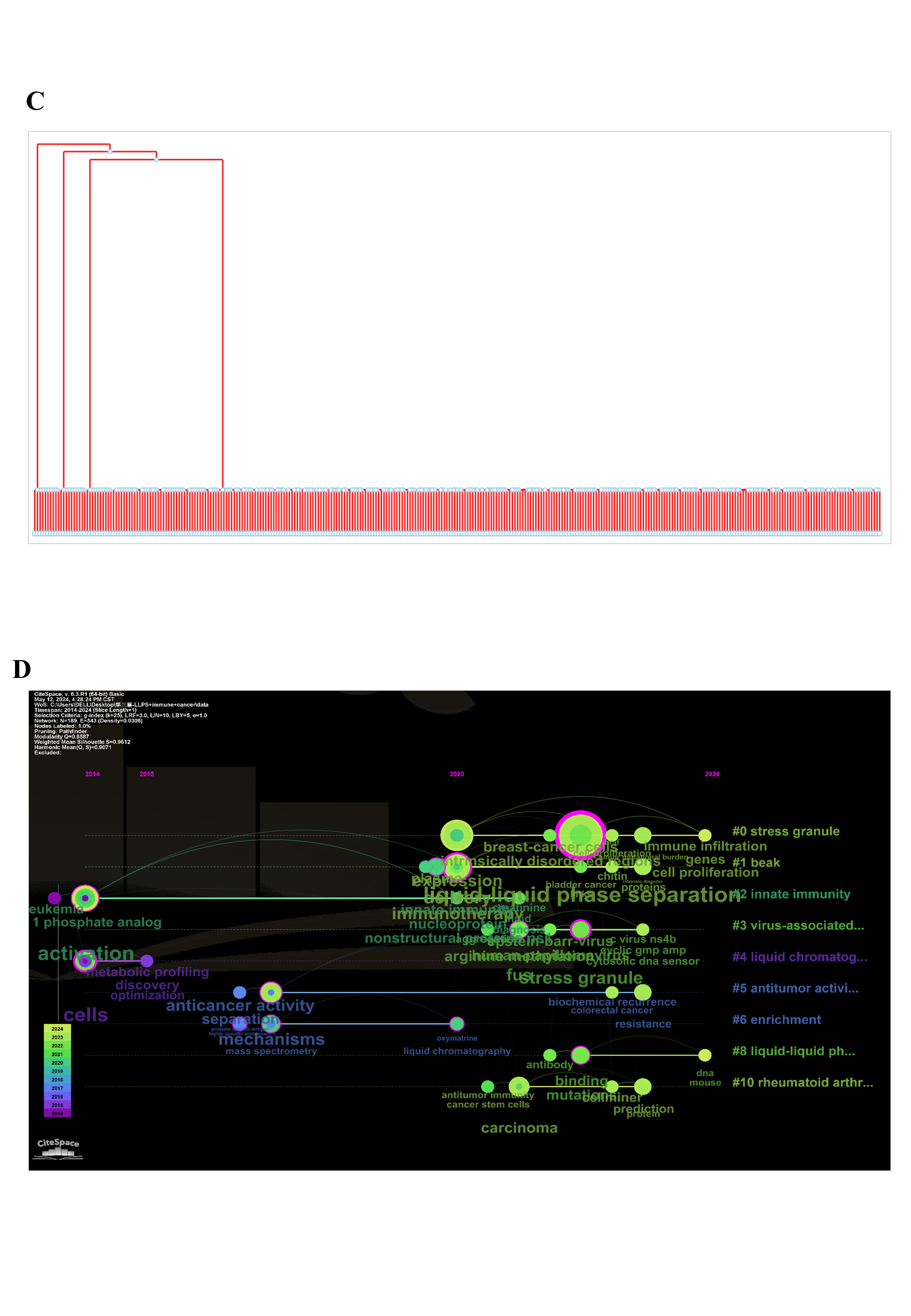

Supplement: Supplementary file 2 [file Image2.tif]
